# Copy Number Variation and Transcriptional Polymorphisms of *Phytophthora sojae* RXLR Effector Genes *Avr1a* and *Avr3a*

**DOI:** 10.1371/journal.pone.0005066

**Published:** 2009-04-03

**Authors:** Dinah Qutob, Jennifer Tedman-Jones, Suomeng Dong, Kuflom Kuflu, Hai Pham, Yuanchao Wang, Daolong Dou, Shiv D. Kale, Felipe D. Arredondo, Brett M. Tyler, Mark Gijzen

**Affiliations:** 1 Agriculture and Agri-Food Canada, London, Ontario, Canada; 2 Department of Plant Pathology, Nanjing Agricultural University, Nanjing, China; 3 Virginia Bioinformatics Institute, Virginia Polytechnic Institute and State University, Blacksburg, Virginia, United States of America; Massachusetts General Hospital, United States of America

## Abstract

The importance of segmental duplications and copy number variants as a source of genetic and phenotypic variation is gaining greater appreciation, in a variety of organisms. Now, we have identified the *Phytophthora sojae* avirulence genes *Avr1a* and *Avr3a* and demonstrate how each of these *Avr* genes display copy number variation in different strains of *P. sojae*. The *Avr1a* locus is a tandem array of four near-identical copies of a 5.2 kb DNA segment. Two copies encoding *Avr1a* are deleted in some *P. sojae* strains, causing changes in virulence. In other *P. sojae* strains, differences in transcription of *Avr1a* result in gain of virulence. For *Avr3a*, there are four copies or one copy of this gene, depending on the *P. sojae* strain. In *P. sojae* strains with multiple copies of *Avr3a*, this gene occurs within a 10.8 kb segmental duplication that includes four other genes. Transcriptional differences of the *Avr3a* gene among *P. sojae* strains cause changes in virulence. To determine the extent of duplication within the superfamily of secreted proteins that includes Avr1a and Avr3a, predicted RXLR effector genes from the *P. sojae* and the *P. ramorum* genomes were compared by counting trace file matches from whole genome shotgun sequences. The results indicate that multiple, near-identical copies of RXLR effector genes are prevalent in oomycete genomes. We propose that multiple copies of particular RXLR effectors may contribute to pathogen fitness. However, recognition of these effectors by plant immune systems results in selection for pathogen strains with deleted or transcriptionally silenced gene copies.

## Introduction

Plant immunity relies on surveillance systems to detect particular infection-specific molecules or perturbations [1], [2]. The timely recognition of a pathogen enables the plant to respond effectively, to prevent the spread of the pathogen beyond the site of infection. Plant pathogens evolve to evade recognition and to suppress plant defenses in order to enhance their fitness and reproductive success. To accomplish this, plant pathogens secrete effector molecules that interfere with plant immune systems or otherwise foster disease [3]. As a consequence, pathogen effector molecules may come under scrutiny by plant surveillance systems and trigger immunity. This process is called effector triggered immunity (ETI). Thus, ETI is a dynamic process that can determine whether disease occurs or not. The genetic components that play a role in this process represent the leading edge in evolution and adaptation in the interaction between the two species, host and pathogen.

Oomycetes are among the most widespread and destructive plant pathogens. The oomycetes resemble fungi in many respects but have evolved independently and are classified separately, in the Stramenopila. Many genera such as *Bremia, Peronospora, Phytophthora,* and *Pythium* cause well-known diseases, but not all oomycetes are plant pathogens. Root rot of soybean caused by *Phytophthora sojae* is a widespread problem that plagues growers and results in annual production losses of 10^9^ kg in North America alone [4]. Global losses far exceed this amount because *P. sojae* occurs in all major growing regions, and soybeans are one of the largest production crops in the world. The integration of *P. sojae* resistance screening into soybean breeding programs is standard practice and the most universal approach to managing the disease. There are two main types of genetically controlled resistance of soybean to *P. sojae*. Partial resistance is mediated by quantitative trait loci (QTL) that lessen the severity of symptoms and curtail the growth of the pathogen [5], [6]. Soybean QTLs for partial resistance to *P. sojae* have been mapped but particular genes have not been identified [7]. Race- and cultivar-specific resistance is controlled by interacting host and pathogen genes that may cause ETI and determine the disease outcome, resistant or susceptible [8]. Soybean *Rps* (*Resistance to P. sojae*) genes and *P. sojae Avr* (*Avirulence*) genes operate as part of the host surveillance and pathogen effector systems, respectively. *P. sojae* races are defined by their ability to infect a set of differential soybean cultivars that carry particular *Rps* genes. Identical race-types may arise independently and thus differ genetically, so this method of classification does not reflect natural phylogeny.

The isolation of the *P. sojae Avr1b*-1 gene, and other oomycete *Avr* genes from *Hyaloperonospora arabidopsis* and *Phytophthora infestans*, has demonstrated that the corresponding Avr effectors are secreted proteins that share a common RXLR (Arg-X-Leu-Arg) motif downstream from the signal peptide [9], [10], [11], [12], [13], [14]. Evidence indicates that the RXLR and associated dEER (Asp-Glu-Glu-Arg; the leading Asp residue is most variable) motifs are targeting elements that deliver the protein effector inside the host cell [15], [16], [17], [18]. Thus, oomycete RXLR effector proteins possess separate protein targeting motifs for secretion and for delivery into host cells, in addition to an effector domain that functions in suppressing host defenses and promoting disease [19], [20], [21], [22], [23]. Additional sequence patterns occurring within the effector domain, called K, L, W and Y motifs, have been identified and suggested to be important functional elements [22], [24]. The RXLR effectors constitute huge families of rapidly evolving proteins in the genomes of *Phytophthora* species [21], [24]. For *P. sojae*, 453 predicted RXLR effectors were catalogued and named Avirulence homolog (Avh) proteins [24].

In this study, we identify *Avr1a* and *Avr3a* from *P. sojae* through independent approaches, relying on genetic mapping and transcriptional profiling. We show how copy number variation and transcriptional differences of these *Avr* genes represent mechanisms for evasion of *Rps* mediated immunity. We also examine the entire family of predicted RXLR effectors from *P. sojae* and *P. ramorum* and provide evidence that multiple, near-identical copies of particular effectors pervade the genomes of these two species. Overall, we suggest that copy number variation of effector genes constitutes a heretofore unrecognized source of genetic adaptation and plasticity for the evolution of oomycete pathogens.

## Results

### Identification of *Avr1a* by map-based cloning

**Figure 1:**
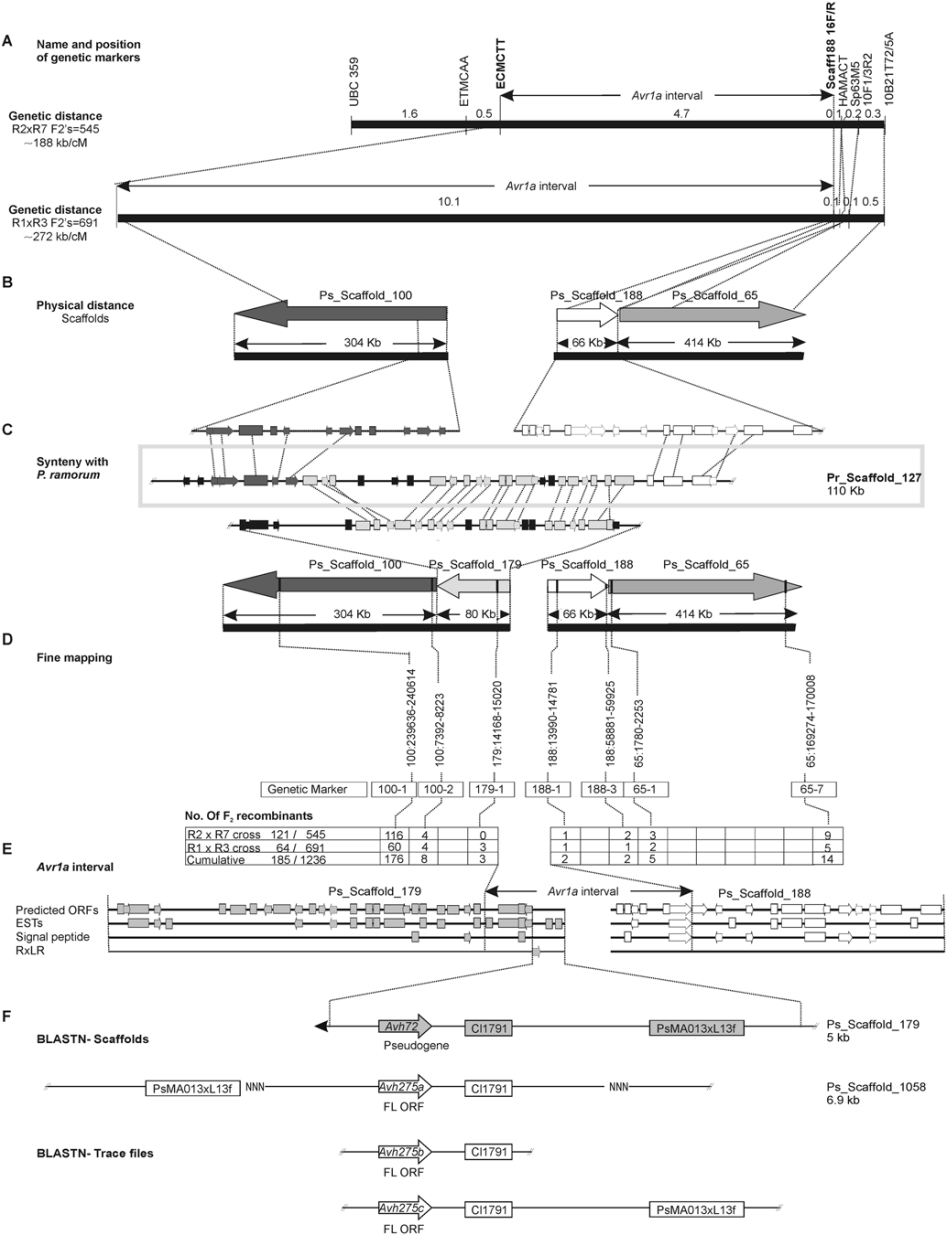
Genetic and physical mapping of the *Avr1a* interval. (A) Genetic map of the *Avr1a* region. The position of *Avr1a* was delimited to a 4.7 cM or a 10.1 cM region, by mapping in separate F_2_ populations. The mapping populations resulted from crosses of different parental strains, as indicated. (B) Physical map of the *Avr1a* locus. Dotted lines link the genetic loci to their appropriate locations on the physical map. Nucleotide sequences corresponding to cleaved amplified polymorphic (CAP) markers ECMCTT, HAMACT, Sp6-3M5, 10F1/3R2 and 10B21T72/5A (Macgregor et al., 2002), were queried against the *P. sojae* draft assembly. This provided anchor points for the integration of the genetic map to sequence contigs. *Avr1a* mapped to an intervening gap between Ps_Scaffold_100 and Ps_Scaffold_188. (C) Conserved synteny of the *Avr1a* locus with *P. ramorum* and identification of Ps_Scaffold_179. Portions of Ps_Scaffold_100 and Ps_Scaffold_188 aligned with opposite ends of a sequence contig (Pr_Scaffold_127) from the *P. ramorum* genome. The intervening sequence of Pr_Scaffold_127 aligned with Ps_Scaffold_179. (D) Fine genetic mapping of *Avr1a*. Analysis of all recombinant individuals from each mapping population place *Avr1a* between DNA markers 179-1 and 188-1. Number of recombinants is given below the respective loci. (E) Genetic features and annotation of Ps_Scaffold_179 and Ps_Scaffold_188. Predicted open reading frames (ORF), expressed sequence tag matches (EST), secreted proteins (signal peptide), and RXLR effectors are shown. (F) The predicted RXLR effector *Avh72* occurs within the *Avr1a* interval. Three additional, nearly identical copies of *Avh72* were discovered, as shown. *Avh275a* occurs on Ps_Scaffold_1058. *Avh275b* and *Avh275c* were assembled from trace files.

Genetic mapping of *Avr1a* was previously accomplished by following segregation of DNA markers and virulence on *Rps1a*, in two different F_2_ populations [25]. Upon the completion of the *P. sojae* genome, we sought to align our genetic maps of *Avr1a* with sequence contig assemblies called scaffolds [26], [27]. Thus, DNA markers in the vicinity of *Avr1a* were mapped to Ps_Scaffold_100, Ps_Scaffold_188, and Ps_Scaffold_65 of version 1.1 of the *P. sojae* genome sequence, as shown in Figure 1A and 1B. Comparison to a syntenic region in *P. ramorum* suggested that an additional contig, Ps_Scaffold_179, occurred in the *Avr1a* region between Ps_Scaffold_100 and Ps_Scaffold_188, as shown in Figure 1C. Further mapping of *Avr1a*, using expanded populations of F_2_ progeny and additional DNA markers derived from the scaffold assemblies, placed *Avr1a* in an interval containing the ends of Ps_Scaffold_179 and Ps_Scaffold_188, as shown in Figure 1D. Within this interval at the end of Ps_Scaffold_179 was a gene encoding a predicted RXLR effector protein, *Avh72* (Figure 1E). Further examination of the *Avh72* sequence revealed that it encodes a pseudogene because the open reading frame (ORF) is interrupted by a premature stop codon. Nevertheless, the *Avh72* sequence was found to share similarity with a predicted RXLR encoding ORF we named *Avh275a* within a small (6,881 bp) contig, Ps_Scaffold_1058. *Avh275a* is 83% identical at the nucleotide level to *Avh72*. An additional paralog to *Avh72*, named *Avh275c* (93% identical to *Avh72*), and another copy of *Avh275a*, named *Avh275b,* were identified by re-assembling trace files from the whole shotgun sequence data. Common features adjacent to or flanking each of the four predicted RXLR effector genes, *Avh72*, *Avh275a*, *Avh275b*, and *Avh275c*, were also found. For example, fragments of a transposon-like element (represented by ESTs Cl1791 and PsMA013×L13f), were associated with each of the RXLR genes (Figure 1F).

**Figure 2:**
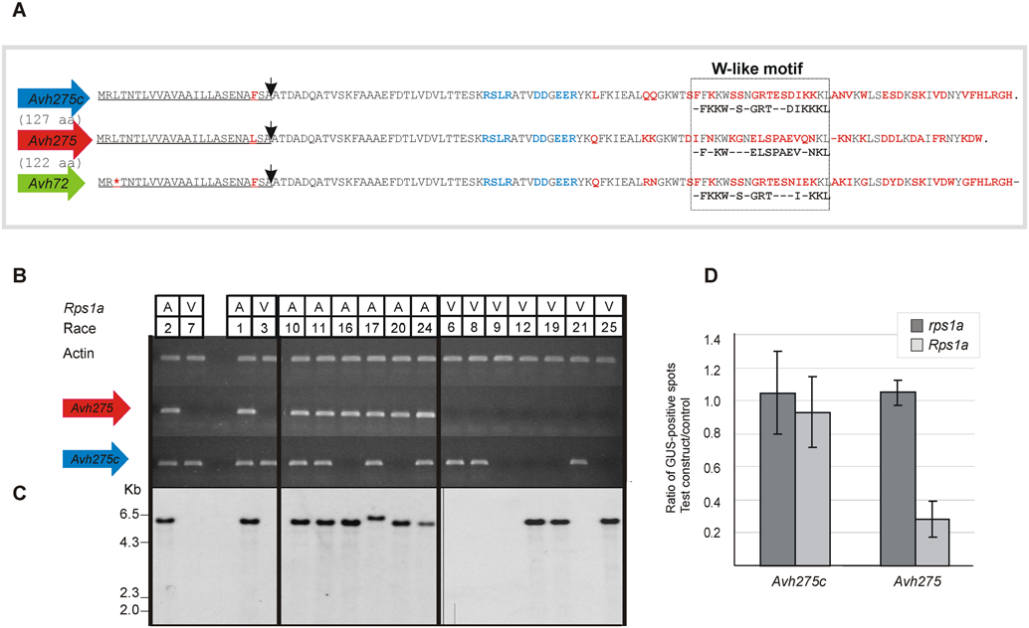
The predicted RXLR effector gene *Avh275* corresponds to *Avr1a*. (A) Primary sequence of Avh72, Avh275 and Avh275c. The signal peptide domain is underlined. Black arrows denote the putative signal peptide cleavage site. Polymorphic amino acid residues are indicated in red. The RXLR dEER motif is shown in blue. The W-like motif is boxed with signature residues outlined below each protein. (B) Expression of the *Avh275* transcript co-segregates with the *Avr1a* phenotype. Shown is an RT-PCR analysis using *Avh275* and *Avh275c* gene-specific primers. A comparison of *P. sojae* isolates that are virulent (V) or avirulent (A) on *Rps1a*; the race designation of each isolate is provided. *P. sojae* actin is shown as a control. (C) DNA blot analyses. Genomic DNA from selected strains were digested with *Kpn*I and hybridized with a probe derived from *Avh275*. Genomic copies of *Avh275* are present in all avirulent races and in the virulent races 12, 19 and 25, but absent in virulent races 6, 8, 9 and 21. (D) Transient expression of *Avh275* triggers an *Rps1a*-specific cell death. Soybean leaves (Harosoy- *rps1a* and Harosoy 63- *Rps1a*) were bombarded with tungsten beads coated with control plasmid pFF19 carrying GUS (pFF19:GUS), or co-bombarded with pFF19:GUS together with test constructs containing *Avh275c* or *Avh275*, as indicated. Signal peptides were retained in the test constructs. Shown is the ratio of GUS-positive spots for the indicated test constructs compared with the control vector. Data represent the mean of at least 14 replicates, and the error bars indicate ± standard deviation.

To determine whether transcripts of the predicted RXLR effector genes could be detected, RT-PCR was performed using template mRNA isolated from *P. sojae* infected soybean. The *Avh275a* and *Avh275b* transcripts are identical to one another and cannot be distinguished (hereafter referred to simply as *Avh275*) whereas *Avh275c* and *Avh72* could be resolved by polymorphisms in the 3′ region (Figure 2A). Each of the predicted protein sequences contained a single W-like motif within the effector domain. No transcripts of *Avh72* were detected, but this was not surprising because it is predicted to be a pseudogene. Transcripts corresponding to *Avh275* were detected, as were transcripts corresponding to *Avh275c*, as shown in Figure 2B. A comparison of *P. sojae* isolates that differed in virulence on *Rps1a* illustrates that *Avh275* is expressed in avirulent races but not in virulent races. Expression of *Avh275c* also differed among the isolates but this did not correlate with virulence to any known *Rps* gene. Southern analysis of *P. sojae* genomic DNA, using an *Avh275-*specific probe, revealed contrasting patterns of hybridization among the isolates, as shown in Figure 2C. Surprisingly, a number of virulent *P. sojae* isolates were found not to possess any copies of this gene as evidenced by a lack of hybridizing fragments. This included the two virulent parents (race 7 and race 3) originally used to generate the F_2_ populations for genetic mapping of *Avr1a*.

The transcriptional and genomic polymorphisms associated with *Avh275* were used as markers to score the F_2_ populations, to determine whether they map to the *Avr1a* region. The results indicate a precise co-segregation of the *Avh275* transcript and genomic copies with *Avr1a* (Figure S1A). Thus, Ps_Scaffold_1058 and *Avh275* genetically map to the *Avr1a* interval and occur in the physical region between Ps_Scaffold_179 and Ps_Scaffold_188. Furthermore, results from haplotype and transcriptional analyses of 17 different *P. sojae* isolates were congruent with the F_2_ mapping data, illustrating that *Avh275* expression is the sole genetic marker that matches *Avr1a* virulence phenotype exactly (Figure S1B).

With all of the evidence suggesting *Avh275* corresponds to *Avr1a*, a functional test for *Avr* genes was performed. This co-bombardment test is based on transient expression of the *Avr* candidate gene with a GUS reporter gene. A positive *Avr* and *R*-gene interaction can trigger cell death and reduce GUS expression, specifically in plant lines that carry the corresponding *R*-gene. Results from co-bombardment tests of *Avh275* and *Avh275c* on soybean plants is shown in Figure 2D. A three-fold reduction in GUS staining was observed for *Avh275* constructs when transiently expressed in *Rps1a* plants. In contrast, expression of *Avh275c* did not reduce GUS expression.

### Identification of *Avr3a* by transcript profiling using microarrays

We hypothesized that gene expression profiles from microarrays could be used in combination with mapping data and whole genome sequence information to rapidly identify *Avr* genes from *P. sojae*. Thus, we used gene chips to find transcriptional polymorphisms associated with *Avr* allelic differences. A pooling strategy, or bulked segregant analysis, was applied to increase screening efficiency and reduce the total number of microarray hybridizations [28], [29], [30]. We profiled gene expression during infection of soybean hypocotyls in the parental isolates, *P. sojae* race 2 and race 7, and compared this to expression patterns in two pools of F_2_ individuals from a cross between the two strains, selected based on their virulence characteristics (Figure S2).

**Figure 3:**
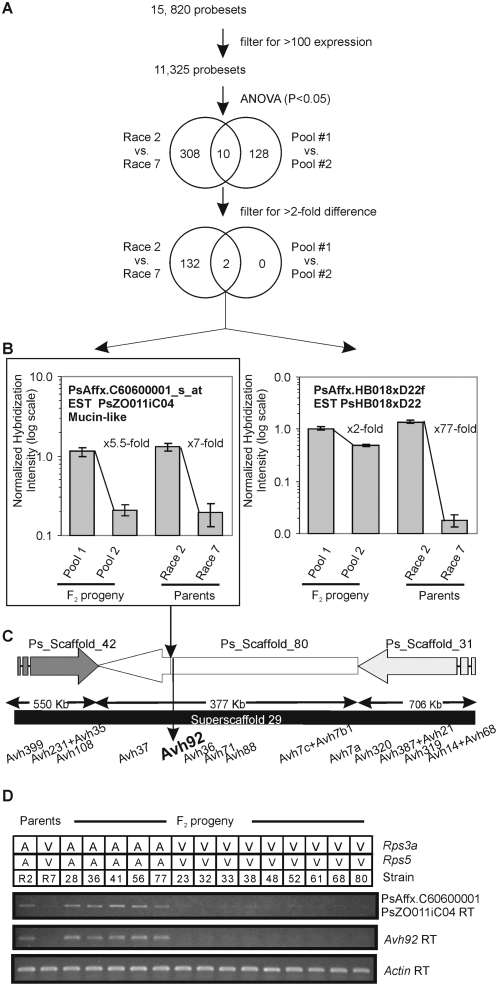
Identification of an *Avr3a*-linked transcriptome marker by array-assisted bulked segregant analysis. (A) Processing of microarray data. The data was filtered to select for probe-sets with raw expression values greater than 100. An analysis of variance (ANOVA, P-value set to 0.05) was performed to identify differentially expressed probe-sets in race 2 (*Avr3a*) compared to race 7 (*avr3a*), and between F2 Pool 1 (avirulent on *Rps3a*) and F2 Pool 2 (virulent on *Rps3a*). Probe-sets identified from the ANOVA analysis were then processed through a filter to select those that displayed a two-fold or greater difference in expression. The Venn diagrams show the number of probe-sets identified at each stage of the analysis. (B) Graphical representation of hybridization data for each of the two probe-sets identified from the microarray analysis. The two probe-sets, PsAffx.C606000001_s_at (EST clone psZO011C04) and PsAffx.HB018×D22f (EST clone psHB018×D22), show greater than two-fold differences in hybridization in each condition (race2 vs. race7, and Pool 1 vs. Pool 2). Each measurement represents the mean and standard error of three independent experiments. (C) The array target PsAffx.C606000001_s falls in a genome region that is rich in predicted RXLR effectors, and lies directly adjacent to *Avh92*. This region of the genome, corresponding to Super-Scaffold 29 (Zhang et al., 2006), contains sequence assembly contigs Ps_Scaffold_42, Ps_Scaffold_80, and Ps_Scaffold_31. The length of each sequence contig (kb) and the position of predicted RXLR effectors (*Avh* genes) are shown. (D) Array target PsAffx.C606000001_s and adjacent gene *Avh92* display similar expression patterns. Shown is an RT-PCR analysis of predicted mucin-like gene (PsAffx.C606000001_s) and adjacent gene *Avh92*. Semi-quantitative RT-PCR analysis was performed on RNA extracted from *P. sojae* parents race 2 (R2) and race 7 (R7) and individual F2 progeny (numbered) originally used in the microarray analysis. The phenotype of each *P. sojae* strain, whether virulent (V) or avirulent (A) on soybean plants carrying *Rps3a* or *Rps5*, is shown. Expression of a *P. sojae* actin gene is shown as a control.

Results from the microarray analysis are shown in Figure 3A. From a total of 15,820 probe-sets on the array, some 11,325 detected transcripts based on signal intensity. To identify transcripts differentially expressed among the parents or the pools, analysis of variance and fold-difference filters were applied. The power of creating pools of F_2_ progeny is clearly evident in the results. Far fewer transcripts are identified as differentially expressed between the pools compared to the parents. Thus, a total of 134 array targets were identified as differentially expressed between the parents whereas only two were different between the pools. The hybridization data for each of these two gene targets is shown in Figure 3B. The predicted sequences for each of these differentially expressed genes did not match any known protein or functional domain, but one (PsAffx.C60600001_s_at) displayed some similarity to a mucin-like protein. This target (PsAffx.C60600001_s_at) mapped to a genome region that is rich in predicted RXLR effectors, as shown in Figure 3C. A close examination of the region reveals that the mucin-like gene lies directly adjacent to a predicted RXLR effector gene, *Avh92*. Two copies each of the array target and *Avh92* are evident in the genome assembly, separated by an assembly gap. There was no probe-set on the array for *Avh92*. To verify the microarray data for the mucin-like gene, and to determine whether *Avh92* shares a similar expression profile, RT-PCR was performed on RNA extracted from samples from the parental strains and the F_2_ progeny used to construct the pools, as shown in Figure 3D. The results show that the mucin-like protein (PsAffx.C60600001_s_at) and the adjacent gene *Avh92* share an identical expression profile. Each of the genes is expressed in race 2 and individual F_2_ progeny in Pool 1, but not in race 7 nor in any of the individual F_2_ progeny of Pool 2.

**Figure 4:**
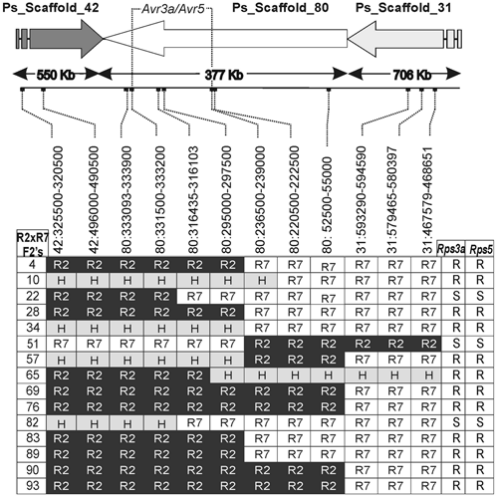
Genetic mapping of the *Avr3a/Avr5* interval. Numbers on the left side indicate informative recombinant F_2_ progeny from a cross of race 2 (R2) and race 7 (R7). The position of genetic markers that were used for mapping is shown, and the genotyping results are presented within the table. Black boxes indicate homozygosity for the race 2 (R2) genotype, white boxes indicate homozygosity for race 7 (R7) genotype, and grey boxes indicate heterozygosity (H). Results from inoculation of each *P. sojae* F_2_ culture on soybean plants carrying *Rps3a* or *Rps5*, is indicated as resistant (R) or susceptible (S).

**Figure 5:**
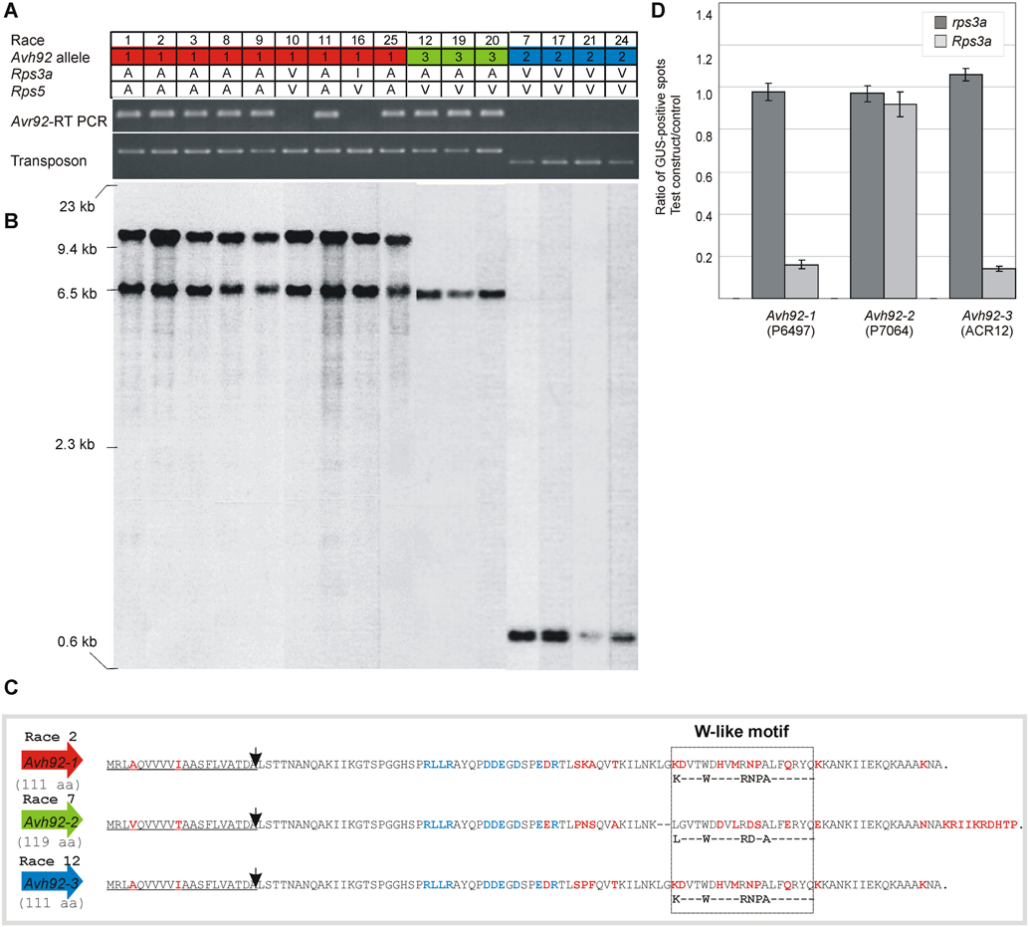
The predicted RXLR effector gene *Avh92* corresponds to *Avr3a*. (A) An RT-PCR analysis of *Avh92* expression among different *P. sojae* isolates is shown, using mRNA template. Also shown is a PCR analysis performed on genomic DNA, to detect a transposon-like sequence polymorphism within the promoter region of *Avh92*. Indicated at the top of the figure is *P. sojae* race tested, the *Avh92* allele present, and the disease outcome, virulent (V), avirulent (A), or intermediate (I), resulting from inoculation on *Rps3a* or *Rps5*. (B) A DNA blot analysis reveals contrasting patterns of hybridization for *Avh92*. Genomic DNA from selected strains were digested with *Kpn*I and hybridized with a probe derived from *Avr92*. Lanes correspond to those shown in (A); the size of DNA markers is indicated on the left. (C) Primary sequence of each of three alleles of *Avh92*. Three different alleles of *Avh92* were identified among *P. sojae* isolates; *Avh92-*1 is present in races 1, 2, 3, 8, 9, 10, 11, 16 and 25; *Avh92-*2 is present in races 7, 17, 21, and 24; and *Avh92-*3 is present in races 12, 19 and 20. The signal peptide underlined. Black arrows denote the putative signal peptide cleavage site. Polymorphic amino acid residues are indicated in red. The RXLR-dEER motif is shown in blue. The W-like domains are boxed with signature residues indicated below each protein (Jiang et al., 2008). (D) Transient expression of *Avh92* triggers an *Rps3a*-specific cell death. Soybean leaves, Williams (*rps3a)* and Williams L83-570 (*Rps3a*), were bombarded with tungsten beads coated with plasmid pFF19 carrying GUS (pFF19:GUS), or with pFF19:GUS together with test constructs containing each of the three alleles of *Avh92*, as indicated. Signal peptides were deleted from test constructs. Shown is the ratio of GUS activity for the test construct compared with the control vector. Data represent the mean of at least three biological replicates, and the error bars indicate ± standard deviation.

Based on the pooling strategy that led to its identification, *Avh92* represented a good candidate for *Avr3a* or *Avr5*. These two *Avr* genes are known to be closely linked or allelic. To determine whether *Avr3a* and *Avr5* map to the vicinity of *Avh92*, DNA markers were designed along the chromosomal region encompassing *Avh92* and scored in 100 segregating F_2_ progeny. Recombinant progeny, shown in Figure 4, indicate that the *Avr3a* /*Avr5* interval corresponds exactly to the region containing *Avh92*. The *Avh92* gene was also examined in a collection of *P. sojae* isolates, to determine whether transcriptional or sequence polymorphisms associate with race-specific immunity conferred by either *Avr3a* or *Avr5*. The *Avh92* transcript was only detected in *P. sojae* isolates that are avirulent on *Rps3a*; virulent isolates do not express the gene (Figure 5A). A polymorphism in the promoter region of *Avh92*, due to insertion of a transposon-like fragment, was also evident in a comparison of *P. sojae* isolates. Southern blot analysis revealed three different RFLP patterns, based upon hybridization to *Avh92* (Figure 5B). Sequencing of genomic DNA indicated that the *Avh92* protein is polymorphic among the isolates, and three different alleles of *Avh92* were identified, as shown in Figure 5C. The most variable region of the protein corresponds to the predicted effector domain, especially in the vicinity of the identified W motif near the C-terminal end.

To test whether *Avh92* interacts with *Rps3a* to activate cell death, transient expression assays were performed by co-bombardment, as shown in Figure 5D. Results show that *Avh92* from *P. sojae* race 2 (P6497) specifically interacts (directly or indirectly) with *Rps3a*, and so this was renamed *Avr3a^P6497^*. The allele from *P. sojae* race 7 (P7064), *Avr3a^P7064^*, did not result in any reduction of GUS intensity by this assay, while the allele from race 12 (ACR12), *Avr3a^ACR12^*, produced a positive interaction as evidenced by reduced GUS intensity. Thus, we conclude that *Avr3a^P6497^* and *Avr3a^ACR12^* are recognized by *Rps3a* but *Avr3a^P7064^* is not.

### *Avr1a* and *Avr3a* display copy number polymorphisms among pathogen strains

**Figure 6:**
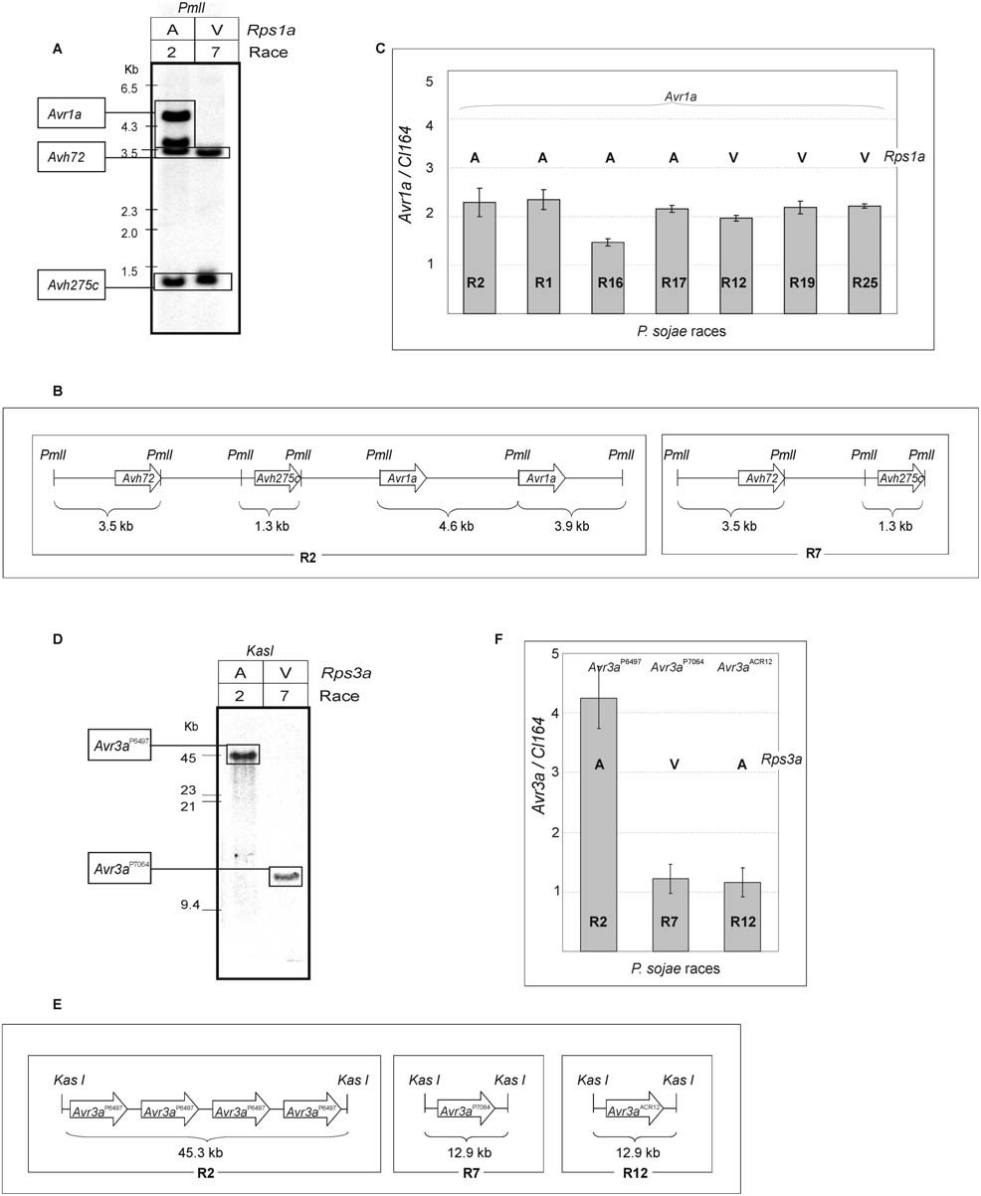
DNA blot analyses and quantitative PCR indicates genomic copy number variation of avirulence genes *Avr3a* and *Avr1a* among *P. sojae* strains. (A) Southern blot analysis of *Pml*I digested genomic DNA from *P. sojae* race 2 (R2) and race 7 (R7); the virulence phenotype on *Rps1a* is indicated as avirulent (A) or virulent (V). The probe, derived from a conserved region, shares sequence identity with *Avh72*, *Avr1a* and *Avh275c.* The identity of each hybridizing band, and the size of DNA standards is indicated on the left. (B) Schematic representation of the *Avr1a* locus in *P. sojae* race 2 (R2) and the corresponding region in *P. sojae* race 7 (R7). Sequences were assembled from trace files and from *de novo* sequence analysis of genomic DNA. The position of *Pml*I sites and the predicted size of hybridizing fragments are indicated. (C) Real-time PCR determination of *Avr1a* genomic copy number among four avirulent (A) and three virulent (V) *P. sojae* races (assayed on *Rps1a*). The *Avr1a* copy number was determined by comparison to single copy gene, *PsCL164*. Data represent the mean and standard deviation from three independent experiments. (D) Southern blot analysis of *KasI* digested genomic DNA from *P. sojae* race 2 (R2) and race 7 (R7); the virulence phenotype on *Rps3a* is indicated as avirulent (A) or virulent (V). The probe corresponds to a conserved region of *Avr3a*. The identity of each hybridizing band, and the size of DNA standards is indicated on the left. (E) Schematic representation of the *Avr3a* locus in *P. sojae* race 2 (R2) and the corresponding region in *P. sojae* race 7 (R7) and race 12 (R12). Sequences were assembled from trace files and from *de novo* sequence analysis of genomic DNA. The position of *Kas*I sites and the predicted size of hybridizing fragments are indicated. (F) Real time PCR determination of *Avr3a* genomic copy number in *P. sojae* race 2 (R2), race 7 (R7) and race 12 (R12) relative to single copy gene, *PsCL164*. The three different *Avr3a* alleles are indicated, and the phenotype on *Rps3a* is shown as avirulent (A) or virulent (V). Data represent the mean and standard deviation from three independent experiments.

**Figure 7:**
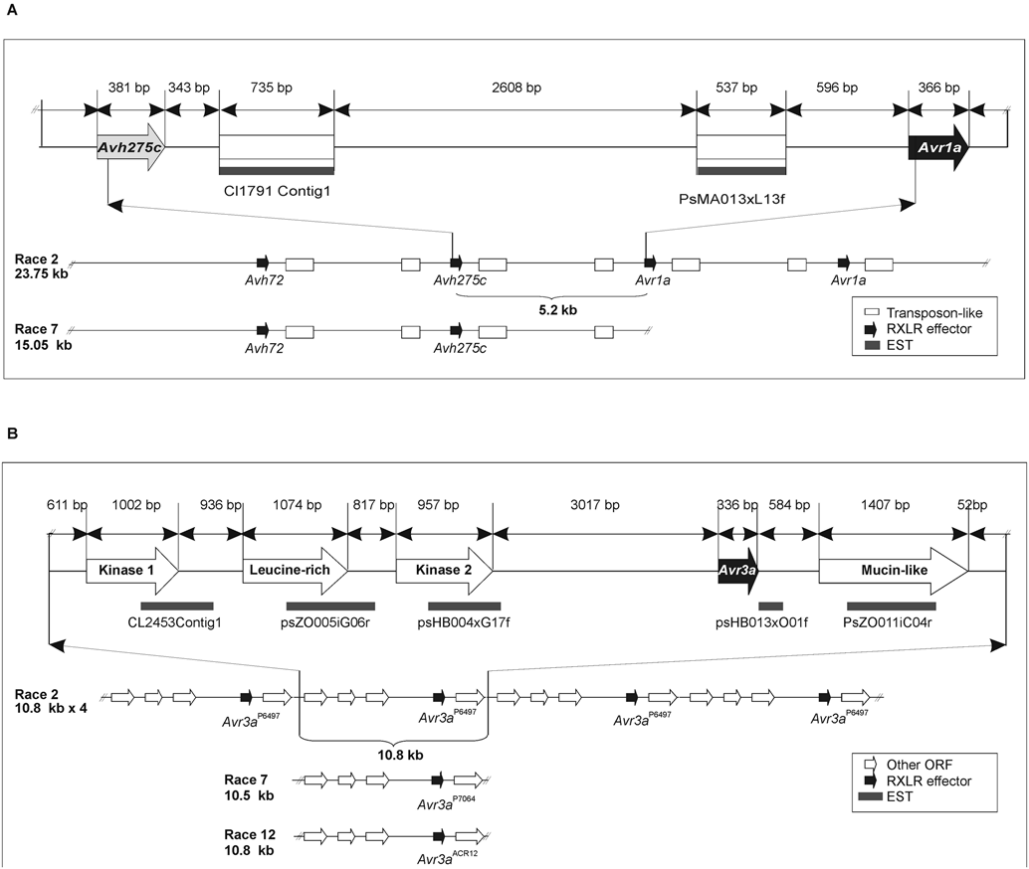
Expansion and contraction of the *Avr1a* and *Avr3a* loci in *P. sojae* strains that differ in copy number. (A) A model for the *Avr1a* locus in *P. sojae* race 2 and race 7. A 23.75 kb segment containing *Avh72*, *Avh275c* and two copies of *Avr1a* is shown for race 2. A corresponding segment of 15.05 kb containing *Avh72* and *Avh275c* is shown for race 7. The 5.2 kb repetitive unit is indicated. A region encompassing *Avh275c* and one copy of *Avr1a* is shown in greater detail, for race 2. Block arrows indicate the position of the open reading frames (ORF) for each gene. Open boxes show the position of closely related or identical transposon-like sequences that match to expressed sequence tags (EST). (B) A model for the *Avr3a* locus in *P. sojae* race 2 and race 7. A 43.2 kb segment containing four copies of *Avr3a^P6497^* is shown for race 2. Corresponding segments of 10.8 kb containing *Avr3a^P7064^* and *Avr3a^ACR12^* are shown for race 7 and race 12, respectively. A region encompassing one 10.8 kb repetitive unit of *Avr3a^P6497^* is shown in greater detail. A total of five predicted open reading frames (ORF) occur on each repetitive unit, as shown by block arrows. The position of matching expressed sequence tags (EST) is shown below the predicted ORF.

The genome sequence assemblies in the vicinity of *Avr1a* and *Avr3a* were not well resolved, a problem typically arising from the presence of repetitive sequences. Further examination, by selective sequencing of genomic DNA fragments, re-assembly of trace files from whole shotgun sequencing, RFLP analysis, and quantitative PCR, led to new sequence models for the *Avr1a* and *Avr3a* loci. These results are summarized in Figures 6 and 7.

The genome assemblies and Southern blot hybridizations for *Avr1a* indicated that multiple related copies of *Avr1a* occur in a cluster in *P. sojae* race 2. Southern analysis after digestion of genomic DNA with *Pml*I could resolve each of these copies, as shown in Figure 6A. Thus, *P. sojae* race 2 contains a gene cluster consisting of the *Avh72* pseudogene, *Avh275c*, and two identical copies of *Avr1a*. Many isolates, such as race 7 possess *Avh72* and *Avh275c* but lack copies of *Avr1a* (Figure 2C and Figure 6B). Copy number analysis of *Avr1a* in isolates containing this gene was also performed by quantitative PCR, as shown in Figure 6C. These results confirmed that two copies of *Avr1a* are present in the genome of *P. sojae* race 2 and most other isolates.

Likewise, multiple identical copies of *Avr3a* are clustered together in *P. sojae* race 2. The *Avr3a* gene cluster could be excised by restriction enzyme digestion (*Kas*I), as shown in Figure 6D. Four identical copies of *Avr3a* occurred on a single 45.3 kb *Kas*I fragment in *P. sojae* race 2, whereas race 7 and race 12 possessed only one copy of *Avr3a* on a 12.9 kb *Kas*I fragment (Figure 6E). This copy number variation was also measured by quantitative PCR using genomic DNA template, as shown in Figure 6F.

Integrating results together from genomic DNA sequencing and RFLP analysis, we were able to re-assemble the *Avr1a* and *Avr3a* loci, as indicated in Figure 7. This shows that the repetitive segment containing *Avr1a* is 5.2 kb and does not include any other genes. Matches of ESTs to intergenic regions of the 5.2 kb repetitive unit likely represent fragments of transposable sequences that may have played a role in the evolution of the locus. In contrast, *Avr3a* occurs in a 10.8 kb repetitive segment along with four other genes. There are four copies of this repetitive segment in *P. sojae* race 2, whereas other *P. sojae* isolates such as race 7 and race 12 possess only one copy.

### *Avr1a* and *Avr3a* genomic regions interrupt conserved synteny with *P. ramorum* and *P. infestans*

**Figure 8:**
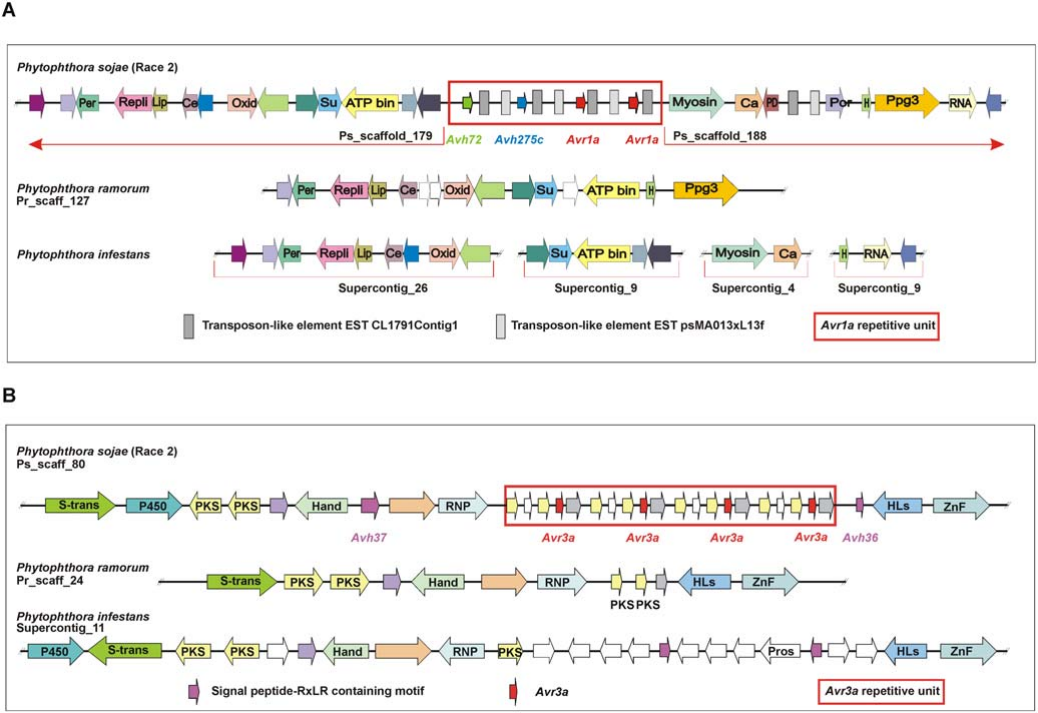
Conservation and interruption of synteny of the *Avr1a* and *Avr3a* ^P6497^ regions in *P. sojae*, *P. ramorum* and *P. infestans.* (A) Comparison of the *Avr1a* region in three different *Phytophthora* species. Colored block arrows indicate the position and transcriptional orientation of putative open reading frames (ORF) with conserved synteny. Similarity to annotated protein sequences is shown, as follows: permease (Per), replication origin activator (Repli), lipoprotein (Lip), cellobiohydrolase A (Ce), sulfite oxidase (Su), sugar fermentation stimulation protein (Su), ATP binding protein (ATP bin), myosin heavy chain (myosin) , Ca^2+^ activated K^+^ channel (Ca), PKD2 related (PD), porphobilinogen deaminase (Por), hypothetical (H), Ppg3 (Ppg3), and RNA helicase (RNA). Open (uncolored) block arrows correspond to predicted ORF without conserved synteny and that lack similarity to known proteins. (B) Comparison of the *Avr3a* ^P6497^ region in three different *Phytophthora* species. Colored block arrows indicate the position and transcriptional orientation of putative open reading frames (ORF) with conserved synteny. Similarity to annotated protein sequences is shown, as follows: sugar transporter (S-transport), cytochrome P450 family protein (P450), threonine kinase-like protein (PKS), EF hand family protein (Hand), RNA-binding protein (RNP), Protease (Pros), ATP-dependent helicase (HLs) and zinc finger protein (ZnF). Open (uncolored) block arrows correspond to predicted ORF without conserved synteny and that lack similarity to known proteins.

A comparative analysis of the *P. sojae Avr1a* and *Avr3a* loci with the corresponding genomic regions in *P. ramorum* and *P. infestans* was performed to determine whether the gene order and structure is conserved among the different species. For the *Avr1a* locus, the flanking regions share similarity in orthologous gene order with segments of sequence contigs from *P. ramorum* and *P. infestans,* as shown in Figure 8A. In *P. ramorum*, this region assembled continuously within a single sequence contig, Pr_Scaffold_127. However, this interval in *P. ramorum* does not encode an *Avr1a* ortholog or any predicted RXLR effectors whatsoever. In *P. infestans*, the region did not assemble continuously and is represented by segments from three different sequence contigs.

For the *P. sojae Avr3a* locus, this region fully assembled within sequence contigs in *P. ramorum* and *P. infestans* (Figure 8B). In *P. ramorum*, there are no *Avr3a* orthologs or RXLR effectors, but the flanking regions and at least three of the five genes from the 10.8 kb repeated segment were recognizable and present in single copy. In contrast, in *P. infestans* there is a large insertion present in the location corresponding to the *P. sojae* locus. This insertion contains many different genes, including two predicted RXLR effectors, but none of these are close *Avr3a* orthologs.

### Depth-sampling reveals multiple copy RXLR effectors are prevalent

To further investigate the repetitive nature of the *Avr1a* and *Avr3a* loci, trace files from whole genome shotgun sequencing were searched to determine the number of matches, and thus the depth of sequence coverage, to particular sequence intervals. Normally, for single copy sequences, the number of matches will be close to the estimated depth of coverage for the overall genome. The *P. sojae* race 2 genome size is 95 Mb, and the total sequence coverage is approximately 9 to 10 fold. More formally, the number of matches *M* to a single copy gene will follow the formula:
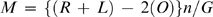
Where *R* = average read length of the trace files, *L* = length of the query sequence, *O* = minimum overlap required to call a match, *n* = the total number of trace file reads, and *G* = genome size. For the *P. sojae* genome, there were a total of 1,533,511 trace files (*n*), with an average length of 937 bp (*R*). Searches using the BLAST algorithm with an expect (*E*) value of 10^−40^ requires an overlap of approximately 100 bp (*O*). Thus, for a query *L* of 100 bp, *M* = 13.5∀7.2 for a single copy gene, where the Poisson distribution predicts the range based upon a 95% confidence interval.

**Figure 9:**
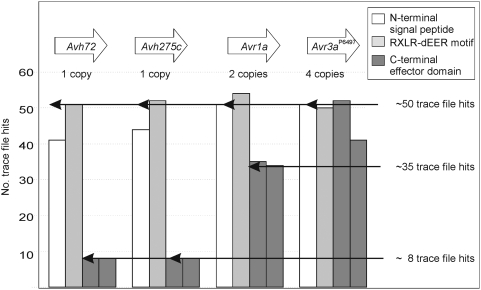
Trace files from whole genome shotgun sequencing provide a means to predict copy number of a sequence segment. The number of trace file matches was determined for segments of *Avh72*, *Avh275c*, *Avr1a* and *Avr3a* ^P6497^. Each open reading frame (ORF) was divided into four segments of approximately 100 bp each. The segments comprised the signal peptides (open bars), the RXLR-dEER motifs (light grey bars), and the effector domains (dark grey bars). Each segment was queried against the *P. sojae* trace files from the whole genome shotgun sequencing (BLAST analysis, E-value cut off of <10^−40^), to determine the number of matches per segment.

The results shown in Figure 9 demonstrate how different 100 bp segments of the *Avh72*, *Avh275c*, *Avr1a* and *Avr3a* genes return varying numbers of trace file matches depending on whether the segment is highly conserved within the repetitive motif. Since most of the 5′ end of *Avr1a* is identical in nucleotide sequence to *Avh72* and *Avh275c*, there are effectively four copies of this segment in the genome and between 40 and 55 trace files matches are returned, values that lie within the expected range of 54∀14. In contrast, the 3′ end of *Avr1a* is only present in each of the two identical copies of the *Avr1a* gene itself, so fewer (34) trace file matches occur, again within the predicted range of 27∀10. For *Avr3a*, there are four identical copies of this gene in *P. sojae* race 2, and thus all segments of the gene return approximately 40 to 50 trace file matches (within the expected range of 54∀14). Thus, trace file depth-sampling potentially offers a powerful and high-resolution means to estimate the copy number of particular sequences.

The contiguous genome regions including the repetitive units that were assembled for the *Avr1a* and *Avr3a* loci were likewise divided into 100 bp segments and queried against the trace file database to provide a depth-sampling profile for each (Figure S3). Predictably, repetitive intervals within the *Avr1a* and *Avr3a* loci are visible in the profile as more deeply sequenced regions. Spikes in this profile represent highly repetitive sequences in the genome, likely corresponding to transposable elements or fragments thereof.

**Figure 10:**
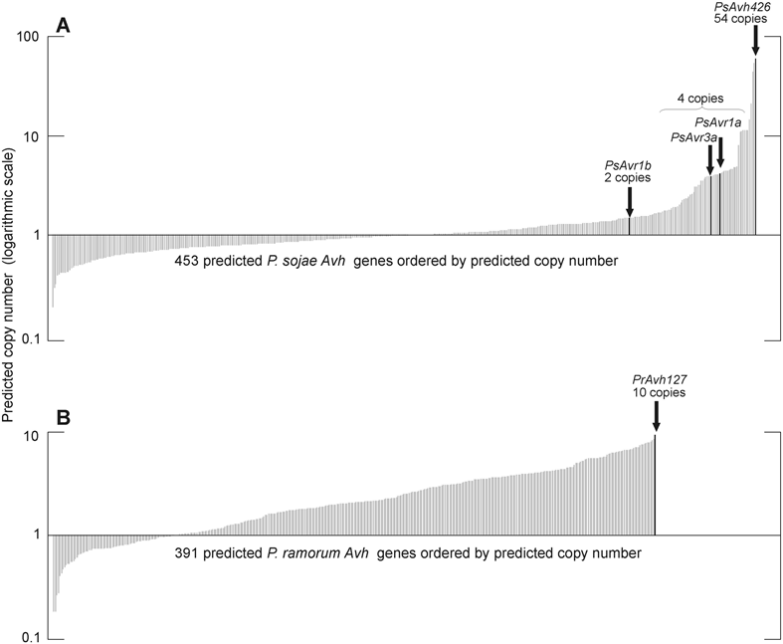
Multiple-copy RXLR effector genes are common in *P. sojae* and *P. ramorum*. (A) Distribution of *P. sojae* RXLR effector genes according to predicted copy number, as determined by depth-sampling of sequence trace files. Selected genes are highlighted for comparison. (B) Distribution of *P. ramorum* RXLR effector genes according to predicted copy, as determined by depth-sampling of sequence trace files. The *Pr_Avh127* gene is predicted to have the greatest number of copies.

The determination of copy number of *Avr1a* and *Avr3a* sequence intervals by depth-sampling was reliable and concordant with results from other methods, such as Southern blot hybridization and real-time quantitative PCR. This encouraged us to investigate gene copy number of the entire RXLR effector family in *P. sojae* by depth-sampling. Results from this analysis are shown in Figure 10A. Sequence intervals corresponding to the complete predicted ORFs of *Avh* genes were used to provide an estimate of the number of copies with a high-degree of sequence identity. The average length of the 453 predicted RXLR ORFs was 557 bp, with a range of 147 bp to 2,748 bp. For *Avr1a* (366 bp) and *Avr3a* (336 bp), we calculate that these genes should match 71∀16 and 69∀16 trace files, respectively, because the four closely related copies comprising the *Avr1a* cluster (*Avh72*, *Avh275c*, and two copies of *Avr1a*), and the four identical copies of the *Avr3a* cluster, should be captured by this analysis. The actual number of hits returned for *Avr1a* and *Avr3a* was 71 and 65, illustrating the accuracy of depth sampling. Similarly, the *Avr1b* gene (417 bp) is known to be represented in the *P. sojae* genome by an additional closely related sequence. Thus, *Avr1b* represents a two-copy RXLR effector, and returns 27 trace file hits, within range of the expected value of 37∀12. Overall, for the complete repertoire of *P. sojae* RXLR effectors, we find that the number of trace file hits per gene, and predicted copy number, varies widely. A total of 77 of 452 (17%) of RXLR effectors are predicted to be present in two or more copies. This suggests that duplicate and multiple genes are common in this family of effectors. The RXLR effector with the greatest depth of sequence coverage and highest predicted copy number corresponded to *Avh426* (375 bp). Based upon the 967 trace file hits to *Avh426*, we estimate that *P. sojae* race 2 contains some 54∀14 near-identical copies of this gene.

The *P. ramorum* RXLR effector super-family was also analyzed by depth-sampling, to provide a comparison to the results from *P. sojae* (Figure 10B). For *P. ramorum*, the average length of the 391 predicted RXLR ORFs was 668 bp, with a range of 180 bp to 2,208 bp. A total of 253 of 391 (65%) of *P. ramorum* RXLR effectors are predicted to be present in two or more copies, a much higher percentage than observed in *P. sojae*. The *P. ramorum* RXLR effector with the highest predicted copy number corresponded to *PrAvh127* (1,725 bp). Based upon the 343 trace file hits to *PrAvh127*, we estimate a copy number of 10∀6. Thus, the *P. ramorum* RXLR super-family displays a narrower range of predicted copy number, compared to *P. sojae*, but yet far more *P. ramorum* effector genes have closely related copies within the genome.

## Discussion

The goal to isolate *Avr* genes has been a major driver in the development of molecular plant pathology. The *Avr* genes have been long-sought because these elements control race-cultivar compatibility and effector-triggered immunity through their interaction with host *Resistance* (*R*) genes. The identification of numerous bacterial and fungal *Avr* genes has revealed a diverse collection of effectors that commonly function to promote disease and suppress plant defenses [31], [32]. For oomycetes, the identification of the first *Avr* genes led to the discovery of new protein targeting motifs that deliver effectors into host cells [12], [33]. Presently, the study of RXLR effectors is drawing attention as researchers try to define the proteins, trace their evolution, and determine how they function and intersect with plant immune systems.

The identification of *Avr1a* and *Avr3a* from *P. sojae* adds to the growing list of oomycete *Avr* genes that are known to encode RXLR effectors, further underlining the importance of this super-family of proteins. However, not all oomycete *Avr* genes are likely to encode RXLR effectors. There is some evidence that the *P. infestans Avr3b-Avr10-Avr11* locus and the proposed *P. sojae Avr1b-*2 locus correspond to transcriptional regulators rather than RXLR effectors [9], [34], [35]. So it is important to continue unbiased approaches to identify new oomycete *Avr* genes. For *P. sojae Avr1a* and *Avr3a*, genetic mapping and transcriptional screening delineated particular genome regions. The recent compilation of predicted RXLR effectors aided our work by providing lists of candidate genes that were often overlooked by gene annotation programs.

Mapping of *Avr1a* to a 30 kb interval required approximately 2,000 F_2_ progeny from two separate crosses, illustrating the relatively low recombination frequency of the region. In contrast, transcriptional screening methods combined with mapping as few as 100 F_2_ progeny resulted in defining the *Avr3a* interval to an 80 kb segment. Each of the genes, *Avr1a* and *Avr3a*, occur as multiple, identical copies in *P. sojae* race 2. Despite that the *Avr1a* and *Avr3a* regions did not assemble properly in whole genome shotgun sequencing, we were able to define a model for each based on further analyses and re-assembly. The structure of the *Avr1a* and *Avr3a* loci and the copy number of the genes themselves vary among *P. sojae* strains. The transcripts are expressed during infection but only in *P. sojae* strains that trigger *Rps-*mediated resistance; strains that are virulent do not express the gene. These transcript differences are due to *Avr* gene deletions or to some form of gene silencing, depending on the *P. sojae* strain.

For *P. sojae* strains that carry copies of *Avr1a* or *Avr3a* but do not accumulate the corresponding transcripts, the mechanism of gene silencing is not known, but past work on the *P. sojae Avr1b-*1 locus may be informative. Characterization of *P. sojae* strains showed that virulent alleles of *Avr1b*-1 were either transcriptionally silent or had accumulated mutations that altered the ORF and evaded *Rps1b* surveillance [9]. Another locus occurring in the vicinity of *Avr1b-*1 was proposed as a transcriptional regulator and was named *Avr1b-*2, but this gene remains unidentified. Likewise, the *P. infestans Avr3b-Avr10-Avr11* locus is unusual and could encode a transcription factor that regulates effector gene expression, but this also needs to be proven [34], [35]. Regardless, it appears that loss of effector gene transcription may be a common mechanism of evading *R-*gene mediated immunity. Conceptually, a switch-regulator that qualitatively controls transcription of effector genes would provide a powerful and useful element for the pathogen in its co-evolutionary struggle with its host.

The present analysis also confirms the linkage of *Avr3a* and *Avr5* in *P. sojae* [36]. In fact, our results suggest the *Avr3a* and *Avr5* may be allelic rather than separate genes. This is supported by mapping data, and by sequence and transcriptional analyses of predicted RXLR effectors occurring in the *Avr3a-Avr5* delimited region. Furthermore, the *P. sojae* race 12, race 19, and race 20 isolates are characterized by differential virulence on *Rps3a* (avirulent) and *Rps5* (virulent). These isolates carry a particular allele, *Avr3a^ACR12^*, that distinguishes them from all other *P. sojae* strains analyzed. Overall, most of the evidence supports the hypothesis that *Avr3a* and *Avr5* are alleles but this has yet to be functionally confirmed.

By examining the genetic space encompassing *Avr1a* and *Avr3a* in *P. sojae,* and comparing this to syntenic regions in *P. ramorum* and *P. infestans*, we found that gene order is largely conserved but is interrupted by insertions and rearrangements in the vicinity of the *Avr* genes themselves. These findings agree with past studies that suggest RXLR effector genes occur in highly dynamic genome regions and are nearly always located at breakpoints that interrupt any conserved synteny [24], [37].

The copy number variation of the *Avr1a* and *Avr3a* genes among different *P. sojae* strains was an unexpected finding, but in retrospect one may have predicted that *Avr* genes would display this kind of genetic polymorphism. Past studies have shown that the *P. sojae* genome contains repetitive sequences that vary from strain to strain [38]. The *P. infestans Avr3b-Avr10-Avr11* locus also shows variation in structure and copy number of the *pi3.4* gene among different strains, although the genetic organization of *pi3.4* has not been fully determined [34]. An appreciation of the prevalence of copy number variation, especially in diploid eukaryotes, has recently emerged as geneticists use new tools and data to track down the genes that control traits [39], [40], [41].

Our analysis demonstrates that multiple, nearly identical copies of RXLR effector genes represent a feature of oomycete genomes that has not previously been recognized. Many of the RXLR effector genes in the *Hyaloperonospora arabidopsis* genome also appear to occur in tandem arrays of diverse sizes (L. Baxter, personal communication). Clusters of identical or nearly identical effector genes have been overlooked because sequence assembly algorithms run into problems in areas of repetitive sequence, especially when the elements occur in adjacent duplicated segments or tandem arrays. Despite the assembly problems they cause, duplicated and repetitive segments of DNA can provide a target for genetic mapping and association studies. Microarrays offer a powerful method for detection of copy number variants by comparative genomic hybridization, but the appropriate platforms need to be available. For species with sequenced genomes, depth-sampling of trace files can offer an alternative method to identify repetitive segments within the genome. These sources of genetic variation can provide important clues or leads for map-based and functional genomic investigations.

Previous studies have shown how the primary sequences of RXLR effectors have been shaped by positive selection, and how the genes themselves undergo accelerated birth and death evolution [21], [24]. Thus, our finding that RXLR effector genes are commonly amplified, and that allelic variation in copy number may underlie changes in virulence, provides further evidence of the rapid evolution of this super-family. We also found differences in the prevalence and extent of amplification of RXLR effector genes by comparing the *P. ramorum* and *P. sojae* super-families. Likewise, past studies have shown that the RXLR super-family of *P. ramorum* has a much higher rate of closely related paralogs than in *P. sojae* [21]. It seems that *P. ramorum* has a larger overall number of recent gene duplications but that particular RXLR effector genes have not been amplified to as high a level as that observed in *P. sojae*. These species-specific patterns have likely been molded by host preferences and degree of specialization, since the RXLR secretome is at the front line in evolution of the host-pathogen interaction [10], [21], [24]. The massive redundancy of particular *P. sojae* genes, such *Avh426* with 54∀14 copies, is likely a direct result of their effectiveness in enabling pathogen growth in soybean. We predict that the specialization of *P. sojae* towards soybean has driven amplification of the high-copy effector genes. Selective pressures shaping the RXLR super-family are bound to be different for *P. ramorum*, since this is a species with a large number of hosts.

Multiple, identical copies of RXLR genes certainly arise from positive selective pressures since these effectors can suppress plant defenses and enhance pathogen growth [19], [20], [22]. Gene amplification is an adaptive mechanism that is inherently unstable, and so it is well-suited to play a role in generating variation in pathogen effector molecules. While the incremental contribution to virulence of a single additional RXLR effector may be difficult to measure against a background expression of hundreds of these genes, natural selection provides a powerful force that shapes genomes on the subtlest of traits. The vast size and the plasticity of the RXLR effector family in *Phytophthora* species suggest a crucial, perhaps indispensable, role in pathogenesis. Nonetheless, particular effectors become a liability rather than an asset when they come under *R*-gene mediated surveillance. Overall, our results illustrate how *P. sojae* adapts to the pressures of a parasitic life through a variety of genetic changes, to meet the challenges imposed by plant immune systems. More specifically, the identification of the *Avr1a* and *Avr3a* genes from *P. sojae* will aid pathogen diagnostics and cultivar development for one of the world's largest crops.

## Materials and Methods

### *P. sojae* isolates

*P. sojae* race 1 (48FPA18) and race 3 (25MEX4), were obtained from the Ohio Agricultural Research and Development Center, Wooster, OH [42]. *P. sojae* race 2 (P6497), race 7 (P7064) and race 19 (P7076), were from the *Phytophthora* culture collection at the University of California, Riverside, CA [43]. All other isolates including races 6, 8, 9, 10, 11, 12, 16, 17, 20, 21, 24, 25, were from the *Phytophthora* species collection at Agriculture and Agri-Food Canada, London, ON (Table S1). Standard procedures were followed for culture and storage of *P. sojae* [44]. Axenic cultures for nucleic acid isolation were prepared by transferring 5 mm mycelial disks cut from the growing edge of each culture to V8 agar plates layered with a piece of cellophane (BioRad). Cellophane sheets overlaid with fully grown mycelial cultures were frozen in liquid nitrogen for later use.

### Expansion of segregating F_2_ mapping populations

F_1_ hybrids were derived from crosses of race 2 (P6497)×race 7 (P7064), and race 1 (48FPA18)×race 3 (25MEX4) [25]. Oospores from F_1_ hybrids were produced by transferring 5 mm mycelial discs cut from the growing edge of a culture to 9 cm 2.5% (v/v) V-8 agar plates supplemented with 10 μg/ml β-sitosterol. Cultures were kept at 25°C in darkness for at least 20 d to produce mature oospores. Oospores were isolated by placing a mature culture in a sterile Waring blender with 100 ml of 4°C SDW. The culture was macerated for 2 min and sieved twice through a sterile 75 μm nylon membrane. The eluant was collected in 50 ml conical tubes and frozen at −20°C to kill hyphae. After 24 hr, the samples were partially thawed at 45°C for 10 min and re-filtered through a sterile 75 μm nylon membrane. The elutant was centrifugated at 3000 rpm for 10 min to pellet the oospores. β-glucuronidase was added to the oospore and water suspension to a final concentration of 2000 U/ml. The mixture was incubated at 37°C for 16 h. Oospores were washed twice prior to resuspending the pellet in 2–5 ml of sterile distilled water. Approximately 500 oospores were spread onto 9 cm 1.5% (w/v) water agar plates supplemented with β-sitosterol (10 μg/ml) and rifampicin (10 μg/ml). Plated oospores were incubated in the dark at 25°C for at least four days before being checked for germination. Germinating oospores were observed using a stereo microscope (60×magnification) and transferred to separate 9 cm 2.5% (v/v) V-8 agar plates supplemented with rifampicin (10 μg/ml). A total of 1,236 F_2_ progeny, including 691 from race 1×race 3 and 545 from race 2×race 7, were produced to establish an expanded mapping population to refine the *Avr1a* interval.

### Plant Material and Disease Assays

Soybean (*Glycine max* (L.) Merr) cultivars Harosoy *(Rps7*), Harosoy 63 *(Rps7, Rps1a*), Haro3272 (*Rps3a*), and Haro34xx (*Rps3c*), Williams, L83-570 (Rps3a), L85-3059 (*Rps5*) and L92-7857 (*Rps3c*), were retrieved from a collection at Agriculture and Agri-Food Canada (Harrow, Ontario) and used as differentials to evaluate the virulence of parental isolates as well as segregating F_2_ populations. Etiolated soybean seedlings were grown in vermiculite soaked in 3 mg /L 15-30-15 fertilizer at 25°C day (16 h) and 16°C night (8 h) temperatures for 7 days prior to harvest for pathogenicity assays. Mycelial plugs (5 mm), cut from the growing edge of 5–7 day old *P. sojae* cultures, were transferred to plant hypocotyls (15–20 per cultivar), mycelial side down roughly 2–3 cm from the base of the cotyledon.

For light grown soybean infection assays, six soybean seeds were sown in 10 cm pots (a minimum of three pots per isolate) containing 3 mg/L 20-20-20 fertilizer soaked soil-less mix (Pro-Mix ‘BX’, Premier Horticulture Ltd, Rivière-du-Loup, Canada). Plants were grown in a controlled growth chamber with supplement light (16 h photoperiod with 25°C day and 16°C night temperatures) prior to biolistic (two week old plants) or virulence assays (one week old plants). *P. sojae* cultures were grown on 0.9% (v/v) V-8 agar plates 5–7 days prior to green plant inoculations. Mycelial inoculums were prepared by passing the actively growing edge of a culture through a 3 ml syringe attached to an 18-gauge needle. Soybean plants were inoculated in the mid-section of each hypocotyl by making a small slit for injection of the mycelial slurry into the wound. Inoculated plants were covered with plastic bags to maintain humidity for two days. Disease symptoms were allowed to develop for an additional period of four days before phenotypes were scored as resistant, susceptible or intermediate. Disease assays were independently repeated at least three times.

### Total RNA isolation, semi quantitative RT-PCR and 5′ and 3′ RACE

Methods for the isolation of total RNA from *P. sojae*-infected soybean tissues have been described [45]. RT-PCR was carried out using the Thermoscript RT-PCR system according to the manufacturers' instructions (Life Technologies, Gaithersburg, MD). Primers-specific for *P. sojae Actin* was used as a control. Full length clones were identified with 5′ and 3′ rapid amplification of cDNA ends from first strand cDNA using the RLM-RACE kit (Ambion 1700, Austin, Tex). Gene specific primers used for RACE work are available in Table S2.

### Genomic DNA preparation, Southern blot analysis and DNA constructs

Genomic DNA was isolated from *P. sojae* mycelial cultures using a modified CTAB (hexadecyl trimethyl ammonium bromide) method [46]. Southern blotting was performed according to standard protocols [47]. Southern blots were hybridized to DIG-labelled probes specific for *Avh275* (115 bp), *Avh275c* (151bp), *Avr3a* ^P6497^ (388 bp), and to a full length *Avh275* probe that hybridizes to *Avh275*, *Avh275c* and *Avh72.* Cloning details of the DNA constructs used in this study are available upon request.

### Linkage analysis and genetic mapping

The *P. sojae* genome assembly version 1.1, arising from race 2 (P6497), was used as a resource for this study, to design primers for amplifying and identifying DNA markers in other strains [26]. Sequence polymorphisms that could be converted into co-dominant markers anchored along the scaffold contig which comprised the *Avr1a* or *Avr3a* locus were screened among 9 virulent and 8 avirulent races as well as F_2_ progeny. A total of 16 cleaved amplified polymorphic (CAPs) markers along *P. sojae* scaffolds 100, 179, 188 and 65, and 15 CAPs markers along scaffolds 42, 80 and 31 were evaluated for linkage with *Avr1a* and *Avr3a*, respectively. Markers described in this manuscript are outlined in Table S3. Conditions for PCR amplification and digestion are available upon request. Virulence phenotypes and DNA marker data from each of the mapping populations were evaluated for linkage map construction using Mapmaker version 3.0 software [48], as described previously [25].

### Transcriptional Profiling of F_2_ Pools

Using an existing cross of *P. sojae* race 2 (P6497) by race 7 (P7064), a total of 100 F_2_ progeny were scored for virulence on *Rps3a*, *Rps3c*, and *Rps5*. Avirulence to *Rps3a* and *Rps5* co-segregated as a dominate trait; avirulence to *Rps3c* segregated independently as a dominate trait. These results replicate past studies [36]. A total of 14 F_2_ progeny were selected to create two pools: Pool 1 contained 5 F_2_ that were virulent on *Rps3c* but avirulent on *Rps3a* and *Rps5*; Pool 2 contained nine F_2_ that were avirulent on *Rps3c* but virulent on *Rps3a* and *Rps5.* Samples of mRNA were purified from each of the parents and 14 F_2_ progeny from the pools, 48 h after inoculation of soybean hypocotyls. Equal amounts of mRNA from each of 5 F_2_ was mixed together for Pool 1, as were samples of mRNA from 9 F_2_ of Pool 2. Integrity, purity and concentration of RNA were verified using an Agilent Bioanalyzer before hybridization to the Affymetrix *Glycine max* GeneChip, a high-density oligonucleotide probe array containing probesets for 15,820 predicted *P. sojae* genes. Three complete biological replicates were performed.

Data was normalized and analyzed using computer software (GeneSpring GX version 7.3). Spot intensity from .cel files was interpreted through an RMA pre-processor. Default normalizations were selected: (1) measurements less than 0.01 normalized intensity were set at 0.01; (2) each chip was normalized to the 50^th^ percentile in comparison with other chips; (3) each gene was normalized by taking the ratio of the raw intensity of a probeset for a particular sample to the median intensity of the probe set calculated across all samples. The normalized hybridization values were plotted as the log of this ratio. Expression profiles for *P. sojae* genes *Actin* and *Avr1b* were visualized to verify the effectiveness of the normalization procedures.

### Sequence analysis and annotation

*P. sojae* trace file sequences were downloaded from the NCBI Trace File Archive (www.ncbi.nlm.nih.gov). Matched trace files and the corresponding mate pairs were manually assembled using computer software (SeqMan, Lasergene 6.0, Madison, WI). Amino acid homology between paralogs of *Avr1a* or alleles of *Avr3a* was determined using the ClustalW algorithm at default parameters using the MegAlign module. Signal peptide searches were performed on predicted open reading frames using the SignalP v.3 software [49]. Sequencing of all constructs and PCR products were performed using dye terminators (BigDye® version 3.1, Applied Biosystems) and a capillary electrophoresis (3130×L Genetic Analyzer, Applied Biosystems). Conserved synteny of orthologous proteins along the *P. sojae* scaffold contig encompassing the *Avr1a* and *Avr3a* loci was compared to *P. ramorum* genome using the PHIGs viewer (www.PHIGs.org). Colinearity with the *Phytophthora infestans* genome was determined by BLAST analysis of the nucleotide sequence of predicted ORFs in *P. sojae* to the *P. infestans* draft genome sequence using the *P. infestans* database developed at the Broad Institute (www.broad.mit.edu).

### Copy number determination using real-time Q-PCR

Gene copy number determinations were made by quantitative PCR (Q-PCR), using an instrument that measures products in real-time (LightCycler®, Roche Diagnostics, Laval, PQ, Canada, software version 3.5). A reference plasmid construct containing *CL164* (one copy per genome):*Avr1a*:*Avr3a* (1:1:1) was used to develop a standard curve with data points ranging from 1 to 10^8^ copies. Primers specific for *Avr1a*, *Avr3a* and *CL164* are available in Table S2. Amplification reactions (20 μl) were performed in duplicate with 106.6 or 1066 pg of input genomic DNA, 3.25 mM MgCl_2_, 0.5 μM of each primer and 2 μl of FastStart DNA Master SYBR Green I mix. PCR parameters were as follows: an initial 10 min- denaturation step at 95°C followed by 40 cycles of 15 s at 95°C, 10 s at 65°C and 12 s at 72°C. Specificity of the primers was verified by a melting curve analysis of the PCR products with a temperature gradient of 0.2°C/s from 68°C to 98°C and by conventional gel electrophoresis. Copy number of *Avr1a* and *Avr3a* was determined as a ratio of the estimated copies of *Avr1a* or *Avr3a* to that of the reference gene, *CL164*.

### Transient expression assays

Co-bombardment assays for *Avr1a* was performed as described [50], [51]. Leaves were photographed using a digital camera. Sample pictures for each leaf were prepared using standard graphic software (Corel PhotoPaint). Samples were obtained by cropping three areas at a fixed size (approximately 1 cm^2^) from each leaf photograph. Unstained leaf material was removed from each photograph using the ‘colour mask’ tool. Remaining GUS stain was analyzed for intensity and area of staining using the ‘volume analysis’ tool from BioRad Gel-Doc imaging software. Particle bombardment assays for *Avr3a* alleles were performed using a double-barreled extension of the Bio-Rad He/1000 particle delivery system as previously described (Dou et al., 2008b). For each paired shot, the logarithm of the ratio of the spot numbers of *Avr3a* to that of the control was calculated, and then the log ratios obtained from the *Rps*3a and non-*Rps*3a leaves were compared using the Wilcoxon rank sum test [52].

### Data deposition

The sequence data of *Avh275*, *Avh275c*, and *Avr3a* have been deposited in Genbank under the accession numbers EF463064, EF467992 and EF587759, respectively.

## Supporting Information

Figure S1 Expression of the Avh275 transcript co-segregates with Avr1a phenotype in F2 progeny and among different P. sojae race types. (A) Segregation analysis of genomic copies of Avh275, and expression of the corresponding transcript, in selected F2 individuals from the mapping population from the race 2×race 7 cross. Genomic DNA was used for PCR analysis to determine the presence or absence of the Avh275 gene. Analysis by RT-PCR was performed on mRNA to determine the presence or absence of the Avh275 transcript. An RT-PCR analysis of a P. sojae actin gene is shown as a control. Indicated at the top of the figure is the P. sojae isolate tested and the disease outcome, virulent (V) or avirulent (A), resulting from inoculation on Rps1a. . (B) Haplotype analysis along the Avr1a interval. The position of DNA markers along sequence contigs (scaffolds) is shown. Numbers on the left side indicate the race of P. sojae isolate. Disease outcome on Rps1a scored as resistant (R) or susceptible (S) is shown on the right of the figure. In addition to genotyping the DNA markers, the presence or absence of genomic copies of Avh275 and the corresponding transcript were scored in all the P. sojae races shown. Red boxes indicate homozygosity for the P. sojae race 2 genotype, yellow boxes indicate homozygosity for race 7 genotype. The names of the genetic markers are shown at the base of the figure.
(1.63 MB TIF)

Figure S2 Virulence phenotypes of parental lines and individual F2 progeny used for bulked-segregant analysis. Soybean hypocotyls were inoculated with P. sojae and photographs were taken 6 d later. Response of soybean cultivars carrying Rps3a, Rps3c and Rps5, are shown.
(1.70 MB TIF)

Figure S3 Depth sampling of the Avr1a and Avr3aP6497 loci. Intervals of 80 kb of the Avr1a (A) and Avr3aP6497 (B) regions were divided into 100 bp units for BLASTn analysis against P. sojae trace files (E-value cut off of <10-40), to determine the number of matches per unit. Red lines border each repetitive unit spanning 5.2 kb and 10.8 kb for Avr1a and Avr3aP6497, respectively.
(1.09 MB TIF)

Table S1 List of Phytophthora sojae strains used in this study.
(0.31 MB DOC)

Table S2 Oligonucleotide primers used in this study.
(0.34 MB DOC)

Table S3 Summary of genetic markers used in this study.
(0.40 MB DOC)
