# Correction: Copy Number Variation and Transcriptional Polymorphisms of *Phytophthora sojae* RXLR Effector Genes *Avr1a* and *Avr3a*

**DOI:** 10.1371/annotation/2a2adcf8-afbc-4d46-92c6-d543d6b29182

**Published:** 2009-06-01

**Authors:** Dinah Qutob, Jennifer Tedman-Jones, Suomeng Dong, Kuflom Kuflu, Hai Pham, Yuanchao Wang, Daolong Dou, Shiv D. Kale, Felipe D. Arredondo, Brett M. Tyler, Mark Gijzen

The plus/minus symbol appears incorrectly as an upside-down Latin capital letter A in the Results subsection "Depth-sampling reveals multiple copy RXLR effectors are prevalent," as well as in a number of other instances highlighted throughout the article. 

