# Supplementary figures and images for: Copy Number Variation and Transcriptional Polymorphisms of Phytophthora sojae RXLR Effector Genes Avr1a and Avr3a
Source: PLoS One. 2009 Apr 3;4(4):e5066. doi: 10.1371/journal.pone.0005066 (PMC2661136; doi:10.1371/journal.pone.0005066)

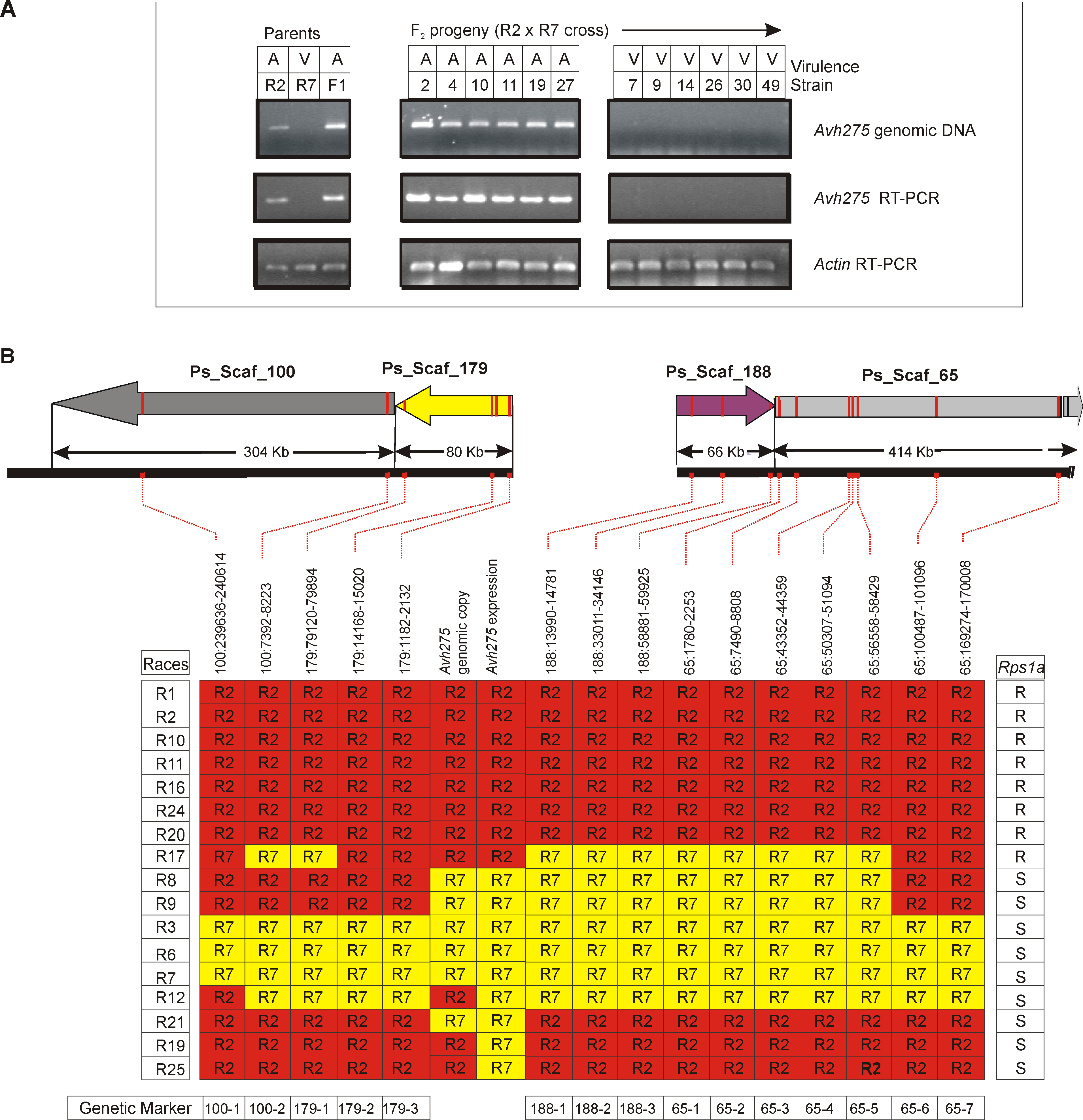

Supplement: Figure S1 — Expression of the Avh275 transcript co-segregates with Avr1a phenotype in F2 progeny and among different P. sojae race types. (A) Segregation analysis of genomic copies of Avh275, and expression of the corresponding transcript, in selected F2 individuals from the mapping population from the race 2×race 7 cross. Genomic DNA was used for PCR analysis to determine the presence or absence of the Avh275 gene. Analysis by RT-PCR was performed on mRNA to determine the presence or absence of the Avh275 transcript. An RT-PCR analysis of a P. sojae actin gene is shown as a control. Indicated at the top of the figure is the P. sojae isolate tested and the disease outcome, virulent (V) or avirulent (A), resulting from inoculation on Rps1a. . (B) Haplotype analysis along the Avr1a interval. The position of DNA markers along sequence contigs (scaffolds) is shown. Numbers on the left side indicate the race of P. sojae isolate. Disease outcome on Rps1a scored as resistant (R) or susceptible (S) is shown on the right of the figure. In addition to genotyping the DNA markers, the presence or absence of genomic copies of Avh275 and the corresponding transcript were scored in all the P. sojae races shown. Red boxes indicate homozygosity for the P. sojae race 2 genotype, yellow boxes indicate homozygosity for race 7 genotype. The names of the genetic markers are shown at the base of the figure. (1.63 MB TIF) [file pone.0005066.s001.tif]

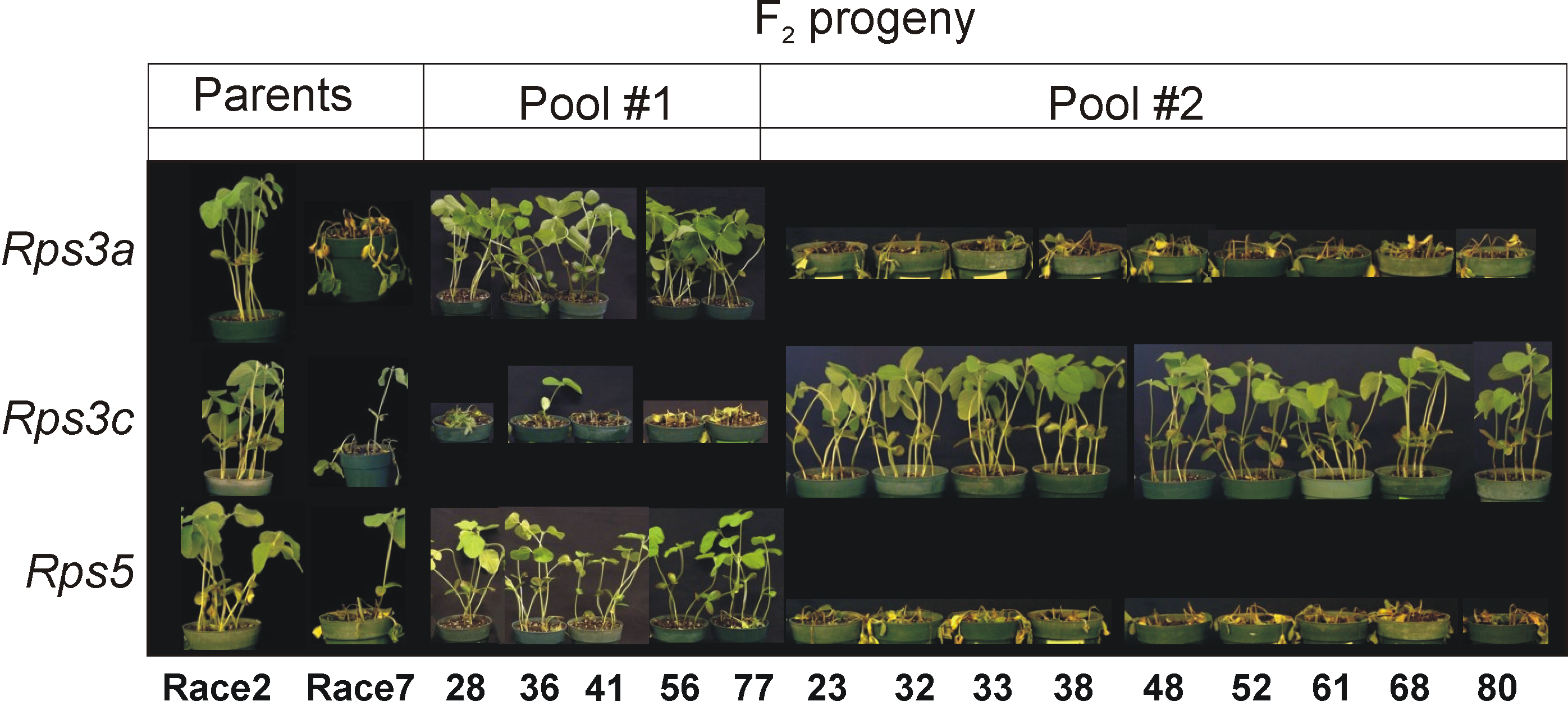

Supplement: Figure S2 — Virulence phenotypes of parental lines and individual F2 progeny used for bulked-segregant analysis. Soybean hypocotyls were inoculated with P. sojae and photographs were taken 6 d later. Response of soybean cultivars carrying Rps3a, Rps3c and Rps5, are shown. (1.70 MB TIF) [file pone.0005066.s002.tif]

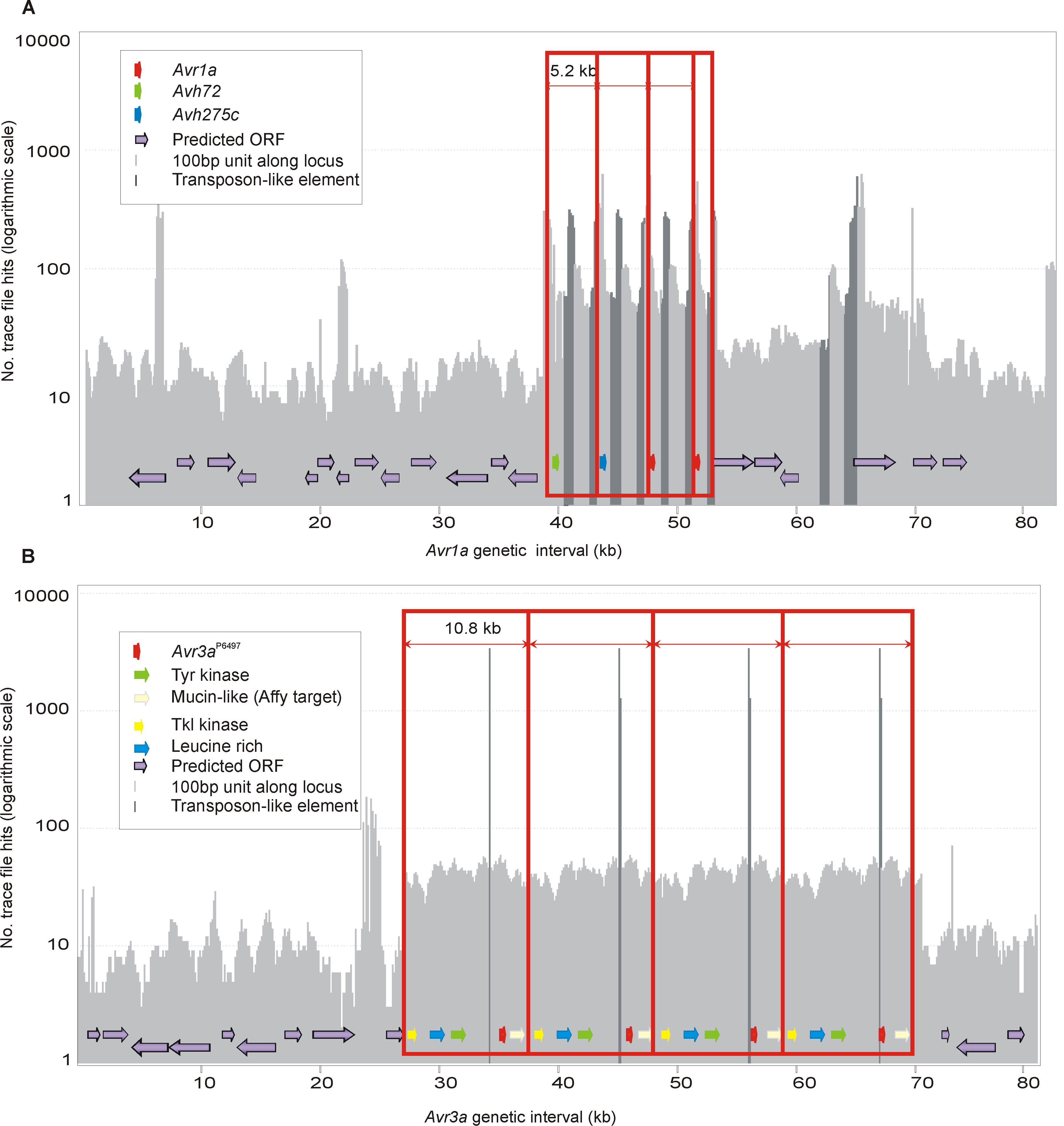

Supplement: Figure S3 — Depth sampling of the Avr1a and Avr3aP6497 loci. Intervals of 80 kb of the Avr1a (A) and Avr3aP6497 (B) regions were divided into 100 bp units for BLASTn analysis against P. sojae trace files (E-value cut off of <10-40), to determine the number of matches per unit. Red lines border each repetitive unit spanning 5.2 kb and 10.8 kb for Avr1a and Avr3aP6497, respectively. (1.09 MB TIF) [file pone.0005066.s003.tif]
