# Supplementary material for: Copy Number Variation and Transcriptional Polymorphisms of Phytophthora sojae RXLR Effector Genes Avr1a and Avr3a
Source: PLoS One. 2009 Apr 3;4(4):e5066. doi: 10.1371/journal.pone.0005066 (PMC2661136; doi:10.1371/journal.pone.0005066)
Supplement: Table S1 — List of Phytophthora sojae strains used in this study. (0.31 MB DOC) [file pone.0005066.s004.doc]

| **Table Sl.** List of *Phytophthora sojae* strains used in this study | | | | | |
| --- | --- | --- | --- | --- | --- |
| Name | Strain | Virulence^a^ | | Origin^b^ | From^c^ |
|  |  | *Rps1a* | *Rps3a* |  |  |
| race 1 | 48FPA18 | A | A | Ohio (F.S.) | F.S. |
| race 2 | P6497 | A | A | Mississippi (B.K.) | B.M.T. |
| race 3 | 25MEX4 | V | A | Ohio (F.S.) | F.S. |
| race 6 | ACR6 | V | V | London, ON (E.W.) | T.A. |
| race 7 | P7064 | V | V | Canada (C.M.) | B.M.T. |
| race 8 | ACR8 | V | A | Unknown | T.A. |
| race 9 | ACR9 | V | A | Harrow, ON (C.M.) | T.A. |
| race 10 | ACR10 | A | V | Stoneville, MS (B.K.) | T.A. |
| race 11 | ACR11 | A | A | Harrow, ON (C.M.) | T.A. |
| race 12 | ACR12 | V | A | Stoneville, MS (B.K.) | T.A. |
| race 16 | ACR16 | A | V | Stoneville, MS (B.K.) | T.A. |
| race 17 | ACR17 | A | V | Stoneville, MS (B.K.) | T.A. |
| race 19 | P7076 | V | A | Stoneville, MS (B.K.) | B.M.T. |
| race 20 | ACR20 | A | A | Unknown | T.A. |
| race 21 | ACR21 | V | V | Lafayette, IN (F.A.L) | T.A. |
| race 24 | ACR24 | A | V | Lafayette, IN (F.A.L) | T.A. |
| race 25 | ACR25 | V | A | Lafayette, IN (F.A.L) | T.A. |
| ^a^Virulence on soybean *Rps* genes; A, avirulent; V, virulent.  ^b^Original site of isolation and where known, investigator who conducted the isolation.  ^c^Investigator who provided the culture to Agriculture and Agri-Food Canada, London, ON. T.A. = T. Anderson (Agriculture and Agri-Food Canada, Harrow, ON); B.K.= B. Keeling (USDA, Stoneville, MS); F.A.L.= F.A. Laviolette (Purdue University, West Lafayette, IN); C.M = C. Meharg (Agriculture and Agri-Food Canada, Harrow, ON); F.S.= F. Schmitthenner (Ohio State University, Wooster, OH); B.M.T.= B. M. Tyler, (Virginia Bioinformatics Institute, Blacksburg, VI); E.W. = Ed Ward (Agriculture and Agri-Food Canada, London, ON). | | | | | |
