# Supplementary material for: Copy Number Variation and Transcriptional Polymorphisms of Phytophthora sojae RXLR Effector Genes Avr1a and Avr3a
Source: PLoS One. 2009 Apr 3;4(4):e5066. doi: 10.1371/journal.pone.0005066 (PMC2661136; doi:10.1371/journal.pone.0005066)
Supplement: Table S2 — Oligonucleotide primers used in this study. (0.34 MB DOC) [file pone.0005066.s005.doc]

| **Table S2.** Oligonucleotide primers used in this study. | | | |
| --- | --- | --- | --- |
| Target gene | Primer name | Sequence, 5’ to 3’ | Application |
|  |  |  |  |
| *Cl164* | CL164Fa | TGGCTTGGCTGTCGATCCT | Real time PCR |
|  | CL164Ra | GTCGGCTCCTCGTACTTCT |  |
|  |  |  |  |
| *Avr1a* | Avr1a R2 | GTCTTTCAAATCGTCGCTCAATT | Real time PCR |
|  | ATG Avr1a-F | CAGTTATCAAGAGCCCGACCA |  |
|  |  |  |  |
| *Avr3a* | Sp92-33F | GCTGCTTCCTTCCTGGTTGC | Real time PCR |
|  | Sp92-325R | GCTGCTGCCTTTTGCTTCTC |  |
|  |  |  |  |
| *Avr1a* | FLAvr1a pFF19-F | ATCGGATCCATGCGCCTAACCAACACCCTCGTC | Primers for cloning full length *Avr1a* into |
|  | Avr1a pFF19-R | ATTGTCGACATGCGCCTAACCAACACC | *BamHI/SalI* site into biolistics construct pFF19 |
|  |  |  |  |
| *Avr1a* | (-SP)Avr1a pFF19-F | GGGGGATCCATGTCTGCAGCAACTGATGCCGA | Primers for cloning *Avr1a*,devoid of its native |
|  | Avr1a pFF19-R | ATTGTCGACATGCGCCTAACCAACACC | signal peptide, into *BamHI/SalI* site of biolistics |
|  |  |  | construct pFF19 |
|  |  |  |  |
| *Avh277c* | FLAvr1a pFF19-F | ATCGGATCCATGCGCCTAACCAACACCCTCGTC | Primers for cloning full length *Avh277c* into |
|  | Avh277c pFF19-R | AAAGCATGCCTAATGACCTCTCAAGTGGAATACATAATT | *BamHI/SphI* site into biolistics construct pFF19 |
|  |  |  |  |
| *Avh277c* | (-SP)Avr1a pFF19-F | GGGGGATCCATGTCTGCAGCAACTGATGCCGA | Primers for cloning *Avh277c* devoid of its native |
|  | Avh277c pFF19-R | AAAGCATGCCTAATGACCTCTCAAGTGGAATACATAATT | signal peptide, into *BamHI/SphI* site of biolistics |
|  |  |  | construct pFF19 |
|  |  |  |  |
| *Avr3a-1* | FLAvr3a-1 pFF19-F | TCTAGATGCGCCTCGCTCAAGTTGTGG | Primers for cloning full length *Avr3a-1* into |
|  | Avr3a-1 pFF19-R | CTGCAGTCGCCGTTGATGATCGGCTA | *XbaI/PstI* site into biolistics construct pFF19 |
|  |  |  |  |
| *Avr3a-1* | (-SP)Avr3a-1 pFF19-F | TCTAGATGCTTTCGACCACGAACGCAAACC | Primers for cloning *Avr3a-1*,devoid of its native |
|  | Avr3a-1 pFF19-R | CTGCAGTCGCCGTTGATGATCGGCTA | signal peptide, into *XbaI/PstI* site of biolistics |
|  |  |  | construct pFF19 |
|  |  |  |  |
| *Avr3a-2* | FLAvr3a-2 pFF19-F | CATTCTAGAGGTACCATGCGCCTCGTTCAAGTTGTGG | Primers for cloning full length *Avr3a-2* into |
|  | Avr3a-2 pFF19-R | CATCTGCAGGGTACCCAGTTTCTGGAGAGAAAACTA | *XbaI/PstI* site into biolistics construct pFF19 |
|  |  |  |  |
| *Avr3a-2* | (-SP)Avr3a-2 pFF19-F | CATTCTAGAATGCTTTCGACCACGAACGCAAACC | Primers for cloning *Avr3a-2*,devoid of its native |
|  | Avr3a-2 pFF19-R | CATCTGCAGGGTACCCAGTTTCTGGAGAGAAAACTA | signal peptide, into *XbaI/PstI* site of biolistics |
|  |  |  | construct pFF19 |
|  |  |  |  |
| *Avr3a-3* | FLAvr3a-3 pFF19-F | ATTGGATCCATGCGCCTCGCTCAAGTTG | Primers for cloning full length *Avr3a-3* into |
|  | Avr3a-3 pFF19-R | ATTGCATGCAGTGTGGTCGCCGTTGATG | *BamHI/SphI* site into biolistics construct pFF19 |
|  |  |  |  |
| *Avr3a-3* | (-SP)Avr3a-3 pFF19-F | ATTGGATCCATGCTATCGACCACGAACGCAAACCA | Primers for cloning *Avr3a-3*,devoid of its native |
|  | Avr3a-3 pFF19-R | ATTGCATGCAGTGTGGTCGCCGTTGATG | signal peptide, into *BamHI/SphI* site of biolistics |
|  |  |  | construct pFF19 |
|  |  |  |  |
| *Avr1a* | Avr1a internal | ATGCGCCTAACCAACACCCTCGTC | Confirmation of the *Avr1a* transgene in *P. sojae* |
|  | Ham34 terminator | AGACACAAAATCTGCAACTTC | transformants |
|  |  |  |  |
| *Avr1a* | Avr1a CE+p35-F | AACGGTACCGACAACAATGCGCCTAACCAACACCCT | Primers for cloning full length *Avr1a* into |
|  | Avr1a CE+p35-R | AAAGGTACCCTACCAGTCTTTATAATTCCTGAA | *KpnI* site of *P. sojae* transformation vector |
|  |  |  | CE+p35 |
|  |  |  |  |
