# Supplementary material for: Copy Number Variation and Transcriptional Polymorphisms of Phytophthora sojae RXLR Effector Genes Avr1a and Avr3a
Source: PLoS One. 2009 Apr 3;4(4):e5066. doi: 10.1371/journal.pone.0005066 (PMC2661136; doi:10.1371/journal.pone.0005066)
Supplement: Table S3 — Summary of genetic markers used in this study. (0.40 MB DOC) [file pone.0005066.s006.doc]

| **Table S3.** Summary of genetic markers used in this study. | | | | | | | | |
| --- | --- | --- | --- | --- | --- | --- | --- | --- |
| Marker^a^ | Scaffold^b^ | Project | Scaffold Position^c^ | Primer | Sequence  5’ to 3’ | Race 2  Marker Size (bp) | Race 7  Marker Size (bp) | Restriction  Enzyme |
| CAP | 3 | *Avr1a* | 3:1240541-1241514 | ETMCAA-F | GTCTGTTGGGGAGCCTGA | 676+298 | 974 | *Eco*RI |
|  |  |  |  | ETMCAA-R | CCGATCCCTGCGCCTCT |  |  |  |
| CAP | 100 | *Avr1a* | 100:239636-240614 | ECMCTT-F | TTGAACAACGTCTCGCTGAT | 491 + 480 | 959 | *Bsi*WI |
|  |  |  |  | ECMCTT-R | CCATGCTGTCCGGCTTGT |  |  |  |
| CAP | 100 | *Avr1a* | 100:7392-8223 | 100-8244-F | CGCCCGGTCGGTCTGTTT | 443 + 240 | 764 | *Sau*3AI |
|  |  |  |  | 100-8244-R | CCGCATTTCAAGTCAAGTCG |  |  |  |
| CAP | 179 | *Avr1a* | 179:79120-79894 | 179-79304-F | GTCGAAGGATATTGGCTGTTT | 401 + 374 | 775 | *Hae*III |
|  |  |  |  | 179-79304-R | CCAGGGGAGTGACAGATGT |  |  |  |
| CAP | 179 | *Avr1a* | 179:14168-15020 | 179-16807-F | ACGGAGACGAGCGGCAAG | 853 | 615 + 212 | *Mse*I |
|  |  |  |  | 179-16807-R | TGGATCATCTCGAGCGTGTA |  |  |  |
| SNP | 179 | *Avr1a* | 179:8861-9859 | 179-7486-F | GCAGTTGGCACAGCGTGTT | CGCTCAG | CGCACAG | - |
|  |  |  |  | 179-7486-R | GTACTGCTCCACCTCCTC |  |  |  |
| CAP | 179 | *Avr1a* | 179:1182-2132 | 179-1182-F | GCACAAGCTAAGGTCAAGAAG | 557 + 478 | 1035 | *Hpa*I |
|  |  |  |  | 179-1182-R | GTAAACCGTGCAGGCACAAA |  |  |  |
| CAP | 188 | *Avr1a* | 188:13990-14781 | 188-16F | CCAAGCTACAGTCACGATCA | 792 | 111 + 678 | *Pst*I |
|  |  |  |  | 188-16R | GTACGGCATTCATGAAG |  |  |  |
| CAP | 188 | *Avr1a* | 188:33011-34146 | 188-Ff | GGATCGCTGAGCTACTACAT | 1136 | 358 + 778 | *Bsm*I |
|  |  |  |  | 188-Fr | GCTCTCTCGCTCAAGGTCT |  |  |  |
| CAP | 188 | *Avr1a* | 188:58881-59925 | HAMACT-F | GATATCGCTTCGGACCTCA | 424 + 621 | 1045 | *Sma*II |
|  |  |  |  | HAMACT-R | AGGAAGAAGAGCCAGTGGT |  |  |  |
| CAP | 65 | *Avr1a* | 65:1780-2253 | 3M5-Sp6-3 | TTCAGACGCAGACACATATTG | 520 + 378 | 350 + 460 | *Nla*III |
|  |  |  |  | 3M5-Sp6-4 | GGCGACGTCACATCTGAA |  |  |  |
| CAP | 65 | *Avr1a* | 65:7490-8808 | 65-Da-F | AGGTGTACGACGCTGTGCT | 261 + 1045 | 1296 | *Xba*I |
|  |  |  |  | DOE4 minus-Ra | CTACACTCACCACCTTACCA |  |  |  |
| CAP | 65 | *Avr1a* | 65:43352-44359 | 65-J-F | GCGTATCCAGCGTGAACTTCA | 1008 | 536 + 460 | *Sma*I |
|  |  |  |  | 65-J-R | GTGGAGCATACGCAGATTCA |  |  |  |
| INDEL | 65 | *Avr1a* | 65:50307-51094 | 65-10/11-F | GAACGCCCACCAACCCATA | 789 | 780 | - |
|  |  |  |  | 65-10/11-R | TACGGCGTGATATCGAACCT |  |  |  |
| CAP | 65 | *Avr1a* | 65:56558-58429 | 10F1 | AGCCCAGCGCTGAATAC | 1551 + 321 | 1872 | *Nco*I |
|  |  |  |  | 3R2 | ACCCGTTTGATGTACAGTGA |  |  |  |
| CAP | 65 | *Avr1a* | 65:100487-101096 | CGA-3 | TGCAGAGTTCAGTCCAACAA | 383 + 227 | 227 + 226 + 166 | *Alw*NI |
|  |  |  |  | CGA-4 | TGCCAGAATTGAGTGGTTAA |  |  |  |
| CAP | 65 | *Avr1a* | 65:169274-170008 | 10B21T7-2 | TCGTCGCCGTATCTTAGG | 520 + 215 | 724 | *Mse*I |
|  |  |  |  | 10B21T7-5A | TCCCCAGCGAGAACAAC |  |  |  |
| CAP | 42 | *Avr3a* | 42: 325500-320500 | R10-3F | CTTGCTCCTTGCGTCTCCTC | 269 + 586 | 855 | *Xba*I |
|  |  |  |  | R10-1R | TACCAACTCGCGCAAGGATC |  |  |  |
| CAP | 42 | *Avr3a* | 42: 496000-490500 | R6-2F | ACCACTGCTGCTCTCTCTCA | 350 + 879 | 1229 | *Aat*II |
|  |  |  |  | R6-1R | GCTTGCATTGAACGAGAACTC |  |  |  |
| CAP | 80 | *Avr3a* | 80:333093-333900 | Avh37S-F | CACACCGACTGGCCAAATC | 666 + 365 + 152 | 894 + 152 | *Hae*III |
|  |  |  |  | Avh37S-R | TGAAGCCTCCTGTTTGGTGT |  |  |  |
| CAP | 80 | *Avr3a* | 80: 331500-333200 | R2-2F | GTGCTGAGCGTGGAGTTACG | 843 | 235 + 608 | *Dpn*II |
|  |  |  |  | R2-2R | GCCGAGATGAATCCCTTGAG |  |  |  |
| CAP | 80 | *Avr3a* | 80:316435-316103 | SP92_33F | GCTGCTTCCTTCCTGGTTGC | 293 | 210 + 77 | *AatII* |
|  |  |  | 80:300039-300374 | SP92_325R | GCTGCTGCCTTTTGCTTCTC |  |  |  |
| CAP | 80 | *Avr3a* | 80: 295000-297500 | NR1-4F | CCGTACGGTATCAGTCTCG | 1048 | 438 + 610 | *Sca*I |
|  |  |  |  | NR1-2R | ACTGCAATGTACCTATGACTTA |  |  |  |
| CAP | 80 | *Avr3a* | 80: 236500-239000 | R8F3 | CAATTACGAGCTCCAACATGT | 693 | 179 + 514 | *Xmn*I |
|  |  |  |  | R8new | TCCATTACAACGCATCTCCTG |  |  |  |
| CAP | 80 | *Avr3a* | 80: 220500-222500 | R7F3 | GCAGACGACGATAATCCTTTG | 290 + 496 | 778 | *Drd*I |
|  |  |  |  | R7R2 | ACCCTCTTGTGCTCTGTATG |  |  |  |
| CAP | 80 | *Avr3a* | 80: 52500-55000 | R3F3 | TTCCCTTCCATCGGCTGTAG | 221 + 550 | 221 + 69 + 481 | *Hha*I |
|  |  |  |  | R3R2 | CCCGGTAATTTATTTCTCTATC |  |  |  |
| CAP | 31 | *Avr3a* | 31: 593290-594590 | Avh320S-F | GTTCGAGGAGCCTATAAAAGTT | 659 + 353 | 1018 | *Cla*I |
|  |  |  |  | Avh320S-R | AGAGAGGTAAAGTAGTGACAAG |  |  |  |
| CAP | 31 | *Avr3a* | 31: 579465-580397 | Avh387S-F | CAAATTTTCGATGTCCGGATAG | 975 | 364 + 634 | *Acl*I |
|  |  |  |  | Avh387S-R | ATGTACGTGGGACCTTTATCG |  |  |  |
| CAP | 31 | *Avr3a* | 31: 467579-468651 | F68pro | CCACTCACGGATCGGCATG | 435 + 251 | 675 | *Rsa*I |
|  |  |  |  | R68pro | TTGAGAGCGAAACGAAAAGTAC |  |  |  |
| ^a^ CAP, cleavage amplified polymorphism; SNP, single nucleotide polymorphism; INDEL, insertion/deletion.  ^b^ Scaffold number based on version 1.1 of the *Phytophthora sojae* genome sequence.  ^c^ Coordinates for the position of the indicated genetic marker along the *Phytophthora sojae* scaffold. | | | | | | | | |
